# Deciphering a missing piece of the branched-chain amino acids uptake puzzle: YhjE is an L-isoleucine and L-valine transporter in *Escherichia coli* K-12

**DOI:** 10.3389/fmicb.2025.1727951

**Published:** 2025-12-03

**Authors:** Sergey V. Molev, Agnessa A. Stepanova, Tatiana V. Vybornaya, Andrey A. Khozov, Makar M. Surkov, Gennadii A. Badun, Maria G. Chernysheva, Alexander I. Netrusov, Alexander S. Yanenko, Sergey P. Sineoky, Dmitrii M. Bubnov

**Affiliations:** 1 National Research Centre “Kurchatov Institute”, Moscow, Russia; 2 Department of Microbiology, Faculty of Biology, Lomonosov Moscow State University, Moscow, Russia; 3 Department of Biophysics, Faculty of Biology, Lomonosov Moscow State University, Moscow, Russia; 4 Department of Radiochemistry, Faculty of Chemistry, Lomonosov Moscow State University, Moscow, Russia

**Keywords:** *Escherichia coli*, L-isoleucine uptake, L-valine uptake, transmembrane transport, amino acid transporter

## Abstract

Branched-chain amino acid (BCAA) uptake in bacteria underpins fitness, pathogenesis, and industrial strain performance, yet the transport landscape, even in *Escherichia coli*, remains incomplete. Here, we identify and characterize YhjE as a BCAA permease specific for L-isoleucine and L-valine. Selection for L-valine–resistant clones in a *ΔlivKHMGF ΔbrnQ* background repeatedly recovered mutations in *yhjE*, indicating that YhjE mediates L-valine entry. Deletion of *yhjE* in the *ΔlivKHMGF ΔbrnQ* background increased the L-isoleucine requirement and raised the minimal inhibitory concentration of L-valine, whereas the L-leucine requirement was unaffected. Direct uptake assays showed that YhjE mediates L-isoleucine and L-valine import. Kinetic analysis yielded apparent K_M_ values of 70.5 μM (L-isoleucine) and 86.7 μM (L-valine), establishing YhjE as a lower-affinity system compared with LIV-I (encoded by the *livKHMGF* operon and *livJ* gene) and BrnQ. Energetically, transport was proton-motive-force–dependent: the protonophore carbonyl cyanide *m*-chlorophenyl hydrazone strongly inhibited uptake, and L-isoleucine addition elicited proton influx as observed by pH measurements. Analysis of transcriptional regulation revealed strict dependence of *yhjE* promoter activity on Lrp and repression by L-leucine, L-methionine, and L-isoleucine, indicating nutrient-responsive control. Hydroxylamine mutagenesis yielded *yhjE* variants that permitted growth under L-leucine limitation; across tested alleles, L-leucine uptake increased while L-isoleucine uptake decreased, demonstrating reprogramming of substrate preference via discrete substitutions. Finally, a *ΔlivKHMGF ΔbrnQ ΔyhjE* triple mutant showed no detectable uptake of L-isoleucine and L-valine at substrate concentrations up to 100 μM, implying that if other routes exist, their contribution at physiological substrate levels is negligible.

## 1 Introduction

Amino acid transport is central to bacterial physiology and to many applications that exploit or combat bacteria. Among these systems, branched-chain amino acids (BCAAs; L-valine, L-leucine, L-isoleucine) are key nutrients whose import is tightly regulated and tightly linked to fitness (Quay and Oxender, 1976; Kaiser et al., 2015; Kaiser and Heinrichs, 2018). In industrial microbiology, membrane transport is a first-class engineering lever: overexpression of efflux systems and, conversely, attenuation or deletion of importers can alleviate product reuptake, reduce feedback inhibition, and redirect flux—thereby improving titers, rates, and yields of BCAA–producing strains (Park et al., 2007; Xie et al., 2012). In pathogenic species, disruption of BCAA uptake attenuates virulence—an observation that motivates transporter-centered antimicrobial strategies and live attenuated vaccine designs (Palace et al., 2014; Dutta et al., 2022). Despite this importance, the inventory and logic of BCAA uptake in bacteria—even in *Escherichia coli*—remain incomplete.

For several decades, it was believed that a branched-chain amino acid (BCAA) uptake machinery of *Escherichia coli* consists of two major active transport systems – LIV-I/LS and LIV-II (Rahmanian et al., 1973). The high-affinity LIV-I/LS system is encoded by the *livKHMGF* operon and the separate *livJ* gene (Adams et al., 1990) and belongs to the ATP Binding Cassette (ABC) Superfamily of transporters. The LIV-I (LivJFGHM) and LS (LivKFGHM) systems share membrane and ATP-binding components, but include distinct substrate-binding subunits (LivJ and LivK) located in the periplasm (Adams et al., 1990). LivJ exhibits affinity for L-isoleucine, L-valine, L-leucine, L-alanine and L-threonine while LivK specifically binds isomers of L-leucine (Antonucci et al., 1985; Khozov et al., 2023). Additionally, the LIV-I and LS systems had been found to be responsible for L-phenylalanine and L-tyrosine uptake with lower affinity than that for BCAA (Koyanagi et al., 2004). The LIV-II transport system is known to have lower affinity toward BCAA than LIV-I and is independent of periplasmic substrate-binding proteins (Rahmanian et al., 1973). A specific gene responsible for the activity of LIV-II, BrnQ, has been identified, cloned and characterized in *Salmonella enterica* (Ohnishi et al., 1988). However, BrnQ of *E. coli,* while a close homolog of *Salmonella enterica* BrnQ has never been associated with the LIV-II activity via direct experiments. The energy-coupling mechanism of BrnQ also remains unresolved; by contrast, BraB, a BrnQ homolog from *Pseudomonas aeruginosa* PAO, utilizes a Na^+^-symport mechanism (Hoshino et al., 1990).

Meanwhile, convincing evidence of the presence of additional uptake systems for BCAA exists in the literature. Using genetic and uptake kinetics data, different authors report the identification of several transporters whose biochemical properties, expression regulation pattern, and energy-coupling mechanism allow to distinguish them from LIV-I and LIV-II (Iaccarino et al., 1978; Yamato et al., 1979). However, structural genes of these permeases remain unknown. In the current work, we provide evidence that at least one of the previously observed activities is associated with the *yhjE* gene, which encodes a membrane protein belonging to The Metabolite: H + Symporter (MHS) Family (Saier et al., 2016). Our results suggest that YhjE is a low-affinity permease specific toward L-isoleucine and L-valine, but not L-leucine, operating as proton-motive-force-dependent symporter. Despite its lower affinity, YhjE activity toward L-isoleucine is comparable in magnitude to that of LIV-I and BrnQ. Its expression is strictly dependent on Lrp and repressed by L-leucine, L-methionine, and L-isoleucine. Finally, we found that the triple *livKHMGF brnQ yhjE* mutant exhibits no detectable transport activity toward L-isoleucine and L-valine at concentrations of the substrates up to 100 μM thereby suggesting that *E. coli* expresses no additional unidentified BCAA carriers active at physiologically relevant conditions.

## 2 Materials and methods

### 2.1 Bacterial strains and plasmids

The bacterial strains used in this study were derived from *E. coli* K-12 MG1655. The genotypes of all strains used are listed in Table 1. The strains were constructed using a combination of λRed-mediated recombineering (Datsenko and Wanner, 2000) and P1 transduction (Thomason et al., 2007). The pUC-*yhjE* plasmid was constructed via ligating two fragments. The vector part was obtained by digestion of pUC18 with SmaI. The insert carrying the *yhjE* gene under control of the native promoter was isolated from pBR-*yhjE* (Bubnov et al., 2025) digested with Eam1104I and EcoRI. The insert was blunted with T4 polymerase prior ligation. The selected recombinant plasmid carried *yhjE* in the reverse orientation with respect to *lacZα*.

**Table 1:** Bacterial strains used in this study.

Strain	Genotype	Source
MG1655	F^−^ *λ*^−^ *ilvG^−^ rfb-50 rph-1*	Laboratory collection
B1905	*∆thrBC ∆sstT ∆ttdT:aadA1 ∆tdcBCDE:neo ΔthrP ΔbrnQ ∆livKHMGF*	This study
B2394	*∆sstT ∆tdcBCDE:neo ΔthrP ΔbrnQ ∆livKHMGF*	
B2541	*ΔleuA ΔlivKHMGF ΔbrnQ*	
B2547	*ΔleuA ΔlivKHMGF ΔbrnQ ΔyhjE:cat*	
B2567	*∆sstT ∆tdcBCDE:neo ΔthrP ΔbrnQ ∆livKHMGF ∆yhjE:cat-sacB*	
B2608	*∆sstT ∆tdcBCDE:neo ΔthrP ∆ilvA:aadA1 ΔbrnQ ∆livKHMGF ∆yhjE:cat-sacB*	
B2609	*∆sstT ∆tdcBCDE:neo ΔthrP ∆ilvA:aadA1 ΔbrnQ ∆livKHMGF*	
B2687	*Δ[araC-araBAD]: P_yhjE_-luxCDABE*	
B2690	*∆sstT ∆tdcBCDE:neo ∆thrP ∆brnQ ∆yhjE:cat-sacB*	
B2694	*∆sstT ∆tdcBCDE:neo ∆thrP ∆livKHMGF ∆yhjE:cat-sacB*	
B2708	*∆sstT ∆tdcBCDE:neo ∆thrP ∆ilvA:aadA1 ∆brnQ ∆yhjE:cat-sacB*	
B2709	*∆sstT:att ∆tdcBCDE:neo ∆thrP ∆ilvA:aadA1*	
B2710	*∆sstT ∆tdcBCDE:neo ∆thrP ∆ilvA:aadA1 ∆livKHMGF ∆yhjE:cat-sacB*	
B2712	*∆leuA:tetA*	
B2713	*∆sstT ∆tdcBCDE:neo ∆thrP ∆leuA:tetA ∆brnQ ∆yhjE:cat-sacB*	
B2714	*∆sstT ∆tdcBCDE:neo ∆thrP ∆leuA:tetA ∆livKHMGF ∆yhjE:cat-sacB*	
B2726	*∆sstT ∆tdcBCDE:neo ∆thrP:aadA1*	
B2826	*Δ[araC-araBAD]*: PH_207_*-lacI* -P_A1O3O4_*-ocr-γβexo-*P_L_*-cat-luxCDABE ∆cysE:tetA*	
B2829	*∆tyrA:tetA*	
B2868	*ΔlivKHMGF ∆tyrA ∆aroP Δmtr ΔtnaCAB ∆pheP:tetA*	
B2911	*ΔlivKHMGF ∆tyrA ∆aroP Δmtr ΔtnaCAB ∆pheP ∆tyrP*	
B2915	*ΔlivKHMGF ∆tyrA ∆aroP Δmtr ΔtnaCAB ∆pheP ∆tyrP ∆yhjE:cat-sacB*	
B2933	*∆sstT ∆tdcBCDE ∆aroP:cI-hok-neo ∆pheP ∆yjeM ΔthrP ΔbrnQ ∆livKHMGF ∆yhjE*	
B2936	*ΔlivKHMGF ∆cysE ∆tcyP ΔtcyLN ΔcyuP*	
B2937	*ΔlivKHMGF ∆cysE ∆tcyP ΔtcyLN ΔcyuP ∆yhjE:cat-sacB*	

### 2.2 Media and culture conditions

Bacteria were routinely grown in lysogeny broth (LB) (10 g/L tryptone, 5 g/L yeast extract, and 10 g/L sodium chloride) at 37 °C with shaking at 220 rpm. A solid LB medium was prepared by adding 20 g/L agar to the LB medium. Ampicillin (200 μg/mL), kanamycin (100 μg/mL), spectinomycin (50 μg/mL), and chloramphenicol (20 μg/mL) were added to the medium as needed. Either solid or liquid M9 minimal medium (Sambrook et al., 1989) with 0.2% glucose was used for the phenotype and transport assays. The medium was supplemented with amino acids as indicated in the Results section and figure legends. Phenotypic assays were performed as previously described (Khozov et al., 2023).

### 2.3 DNA manipulations

Standard methods were used for chromosomal DNA isolation, restriction enzyme digestion, agarose gel electrophoresis, ligation, and transformation (Sambrook et al., 1989). PCR amplification was performed using DreamTaq (Thermo Fisher Scientific, Vilnius, Lithuania) or KAPA HIFI (Kapa Biosystems, Wilmington, MA, USA) polymerase. Plasmids were isolated and DNA fragments were extracted using GeneJET Plasmid Miniprep and GeneJET Gel Extraction kits (Thermo Fisher Scientific).

### 2.4 Labeling amino acids with ^3^H

L-[^3^H]valine and L-[^3^H]isoleucine were prepared by the tritium thermal activation method (Badun and Chernysheva, 2023) with conditions guided by Badun et al. (2012). Briefly, aqueous solutions of L-valine or L-isoleucine were evenly distributed on the inner walls of a glass reactor and lyophilized. The reactor was then connected to a gaseous tritium line, evacuated, and filled with tritium. A tungsten filament inside the vessel was heated to 1850K for 10 s while the reactor walls (bearing the amino acid target) were cooled with liquid nitrogen (77K). Following exposure to tritium atoms, the target was dissolved in water. To remove labile tritium, the solution was kept for 24 h and lyophilized; this exchange/lyophilization cycle was repeated twice.

Crude products were purified by thin-layer chromatography (TLC) on silica gel plates (Silica Gel on Aluminium, 20 × 20 cm, Sigma-Aldrich) using n-butanol:acetic acid:water (3:1:1, v/v/v) as the mobile phase. Compound positions were identified by running nonradioactive standards in parallel and visualizing with ninhydrin under identical conditions. Labeled products were eluted from silica with water. Radiochemical purity was assessed by TLC in ethanol:ammonia:ethyl acetate (4:1:5, v/v/v) and was 98% for L-[^3^H]valine and 97% for L-[^3^H]isoleucine. The final preparations had molar activities of 30 TBq mol^−1^ (L-[^3^H]valine) and 14 TBq mol^−1^ (L-[^3^H]isoleucine).

### 2.5 Amino acids uptake assay

L-[^3^H]isoleucine and L-[^3^H]valine were prepared as described above. L-[U-^14^C]leucine was obtained from Moravek Biochemicals (USA). Uptake assays and K_M_ measurements were performed as described previously (Khozov et al., 2023; Bubnov et al., 2025). Briefly, cells were grown overnight in M9 medium with 0.2% glucose at 37 °C (with 100 mg/L ampicillin for plasmid-bearing strains), diluted to OD₆₀₀ = 0.0625, grown to OD₆₀₀ ≈ 0.5, harvested, washed once with M9, and starved for 2 h in M9 + 0.2% glucose to deplete intracellular amino acids. Suspensions were concentrated to OD₆₀₀ = 32, chloramphenicol was added to a final concentration of 50 μg/mL, and cells and radiolabeled substrates were preincubated separately for 20 min at 37 °C. Uptake was initiated by rapidly mixing the pre-warmed cell suspension with the pre-warmed radiolabeled amino acid solution at 37 °C to obtain a final OD₆₀₀ of 10; both cells and substrates were in M9 medium with 0.2% glucose. At specified time points, 12.5 μL aliquots were vacuum-filtered through 0.45-μm nitrocellulose, washed twice with M9, the membranes were dried, and radioactivity was quantified by liquid scintillation counting. Cell-free controls matched for each substrate concentration were processed identically and subtracted. Transport rates were expressed as nmol mg^−1^ DCW min^−1^ using an OD₆₀₀-to-DCW conversion as in Khozov et al. (2023).

### 2.6 Measurement of *in vivo* luminescence and bacterial growth

A single colony of the assayed strain was inoculated into 5 mL of LB medium. The overnight culture was diluted to an initial OD_600_ of 0.004 with fresh M9 medium supplemented with 2 g/L glucose. Two hundred microliters of the culture were added to each well of a 96-well plate (black-walled, transparent flat bottom; cat. #665096 Greiner Bio-One, Frickenhausen, Germany). The outer wells were not used to avoid edge effects. A well containing a sterile medium was used as a blank. The plates were incubated at 37 °C with double-orbital shaking at 600 rpm using a CLARIOstar Plus luminometer (BMG Labtech, Ortenberg, Germany), and OD_600_ and luminescence were measured every 15 min. No luminescence emission filter was used. The photomultiplier gain was automatically controlled using an enhanced dynamic range function. The measured values were normalized to a 1 s accumulation time. The acquired data were analyzed using the MARS software. Blank values were subtracted from the raw OD_600_ and relative luminescence units (RLU) values. The corrected RLU reads at each time point were divided by the corresponding OD_600_ values to normalize the RLU per cell mass for each well. The average RLU/OD_600_ values and standard deviations were calculated and plotted against the OD_600_.

### 2.7 Structural modeling and visualization

The protein structures predicted using AlphaFold 2 (Jumper et al., 2021) were obtained from the AlphaFold Protein Structure Database (Varadi et al., 2022). Structural visualization was performed using UCSF ChimeraX (Pettersen et al., 2021).

### 2.8 Energetic coupling assay via pH measurement

Cells were cultured overnight in a rich medium (g/L: NaCl, 2.5; K₂HPO₄, 2.5; yeast extract, 35; glucose, 2.5; pH 7.2) with the required antibiotics. The cultures were diluted 1:15 with the same medium, and the incubation was continued for an additional 1.5–2 h. The cells were pelleted by centrifugation at 5000 × g, washed, and resuspended in a minimal medium (34 mM KH_2_PO_4_, 64 mM K_2_HPO_4_, 20 mM (NH_4_)_2_SO_4_, 300 μM MgSO_4_, 1 μM FeSO_4_, 1 μM ZnCl_2_, 10 μM CaCl_2_) supplemented with 10 g/L glycerol as a carbon source. Cells were incubated for 3.5 h at 37 °C with shaking at 220 rpm for YhjE induction. Next, the cells were pelleted by centrifugation at 5000 × g, washed twice with buffer (120 mM choline chloride; 2 mM MgSO₄) and resuspended in the same buffer. The cell suspension was adjusted to an OD₆₀₀ of 25. The transport coupling assay was performed at 25 °C in a 15 mL vessel with a constant supply of gaseous nitrogen and vigorous stirring, using a pH electrode connected to a SevenDirect SD20 pH meter (Mettler Toledo, Switzerland). Once the pH of the cell suspension had stabilized, L-isoleucine (pH 7.25) was added to a final concentration of 1 mM, and the resulting pH changes were continuously recorded.

### 2.9 Plasmid *in vitro* mutagenesis and screening of YhjE variants with altered substrate specificity

Two micrograms of the pBR-*yhjE* plasmid DNA were incubated for 1.5 h at 75 °C in 200 μL of 50 mM potassium phosphate buffer (pH 5.8) containing 400 mM hydroxylamine hydrochloride (Ogawa-Miyata et al., 2001). Aliquots (50 μL) were taken every 30 min and plasmid DNA was purified using the Cleanup Standard kit (Evrogen, Russia) according to the manufacturer’s instructions. The purified plasmid was transformed by electroporation into the L-leucine auxotrophic B2547 strain lacking all known transport systems for this amino acid. Transformants were selected on M9 minimal agar supplemented with 200 μg/mL ampicillin and 2 mg/L L-leucine. Plasmid DNA was isolated from the resulting transformants and retransformed into the same strain. Transformants were selected on LB agar supplemented with 200 μg/mL ampicillin and subsequently screened for growth on M9 agar with 2 mg/L L-leucine. Variants that enabled the growth of the B2547 strain on minimal medium at a limiting concentration of L-leucine were isolated. The resulting plasmids were sequenced.

## 3 Results

### 3.1 YhjE is a low-affinity transport system specific toward L-isoleucine and L-valine

To identify additional systems involved in BCAA import, we exploited an intrinsic regulatory property of *E. coli* acetohydroxyacid synthases (AHAS) IlvIH and IlvBN, which are feedback-inhibited by L-valine (Vyazmensky et al., 2009). This inhibition causes L-isoleucine starvation and thereby renders L-valine toxic to *E. coli* K-12 grown on minimal medium. Spontaneous L-valine–resistant mutants of the B1905 strain, which lacks the major L-valine permeases LIV-I and BrnQ, were selected on M9 medium supplemented with 0.2% glucose and 20 mg/L L-valine, and their genomes were subsequently resequenced. Strikingly, six out of 20 independently selected mutants carried nucleotide changes mapping to the *yhjE* open reading frame that resulted in the F84L, G282E, G415R, P125L, G65L, and V298G substitutions in the YhjE polypeptide (for nucleotide substitutions see Supplementary Table S1). None of the recovered *yhjE* alleles was an obvious loss-of-function; nevertheless, the recurrence and distribution of these changes are most parsimoniously explained by partial reduction of YhjE activity, which would be expected to decrease membrane permeability to L-valine and thereby confer resistance.

This genetic observation is complemented by our previous functional data on YhjE. We previously showed that *yhjE*, when present on a multicopy vector, complements L-threonine uptake deficiency in a strain lacking the principal threonine permeases TdcC, SstT, ThrP, LIV-I, and BrnQ (Bubnov et al., 2025). Conversely, inactivation of *yhjE* aggravates the threonine-uptake defect in the same background. Because the known BCAA carriers LIV-I and BrnQ are able to transport both branched-chain amino acids and L-threonine, these observations raise the reciprocal possibility: a protein that functions (even weakly) as a threonine transporter may act as a dedicated BCAA carrier.

Taken together, the enrichment of *yhjE* mutations among L-valine–resistant isolates obtained from the *ΔlivKHMGF ΔbrnQ* mutant, the multicopy complementation of threonine uptake by *yhjE*, and the substrate range overlap of established transporters support the hypothesis that YhjE contributes to cellular uptake of L-valine and, possibly, L-isoleucine and L-leucine.

**Figure 1:**
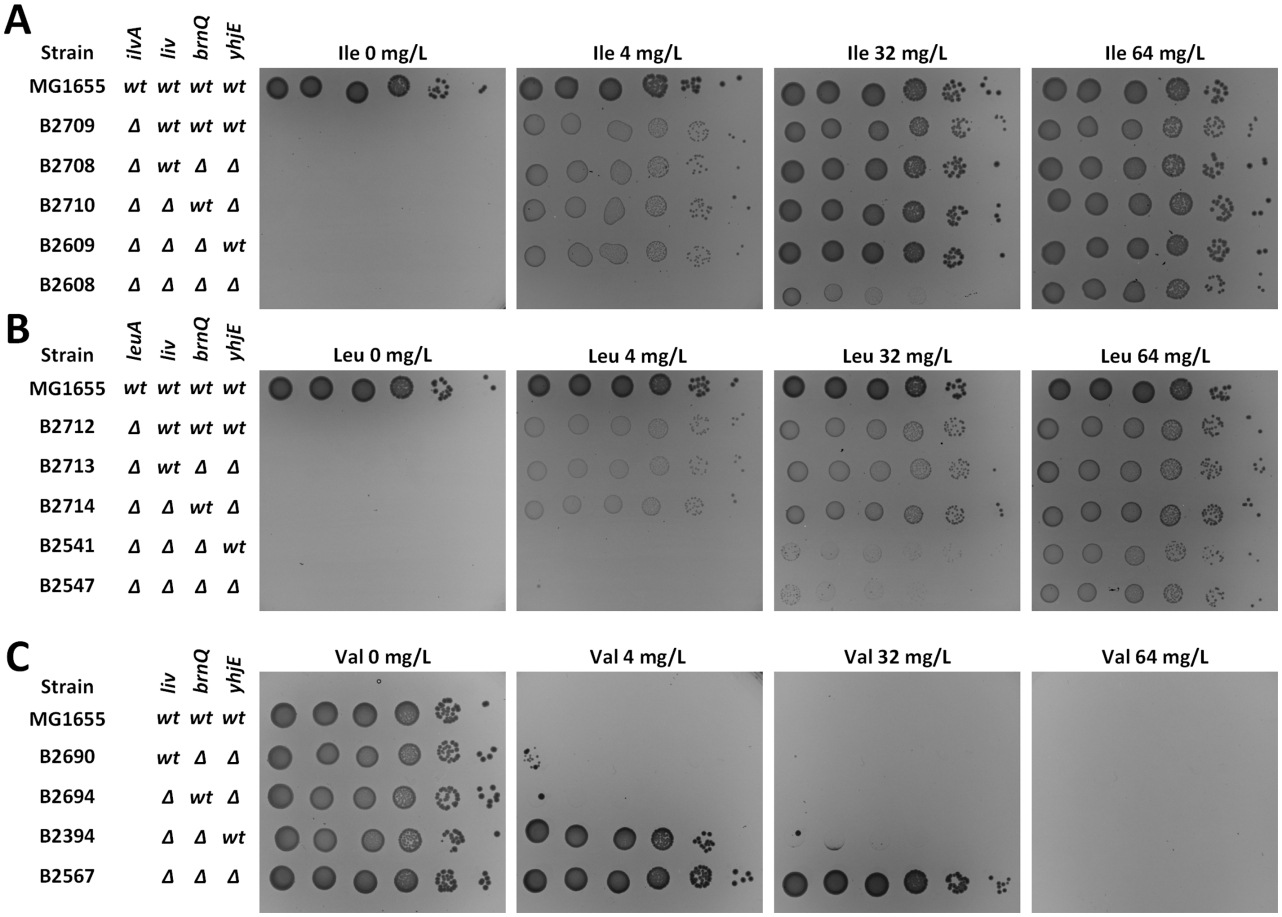
Analysis of the *yhjE* mutation phenotype. Phenotypic assays were performed on M9 minimal medium supplemented with 0.2% glucose and the indicated amino acids. “Ile,” “Val,” and “Leu” denote L-isoleucine, L-valine, and L-leucine, respectively. The genotype of each strain with respect to transport systems and auxotrophies is shown to the left of each panel; “*liv*” denotes *livKHMGF*. Panels illustrate the effect of the *yhjE* mutation on the L-isoleucine requirement **(A)**, the L-leucine requirement **(B)**, and growth inhibition by L-valine **(C)**.

Indeed, we confirmed these results by targeted inactivation of *yhjE* in a strain already deficient in LIV-I and BrnQ. In an L-isoleucine-auxotrophic background, loss of *yhjE* markedly increased the auxotrophic requirement: the *ΔlivKHMGF ΔbrnQ ΔyhjE* triple mutant required 32 mg/L L-isoleucine to support growth in minimal M9 medium, whereas the isogenic *ΔlivKHMGF ΔbrnQ* strain retaining the wild-type *yhjE* allele grew at only 4 mg/L (Figure 1A). This pronounced difference clearly indicates that YhjE contributes to L-isoleucine uptake under these conditions.

We next performed a similar assay using L-leucine-auxotrophic derivatives. In contrast to the L-isoleucine case, the B2547 (*ΔlivKHMGF ΔbrnQ ΔyhjE*) and B2541 (*ΔlivKHMGF ΔbrnQ*) strains displayed identical threshold concentrations for L-leucine (Figure 1B), suggesting that YhjE either does not mediate L-leucine uptake or its contribution is negligible compared with other transport routes active in this background.

Finally, we compared the growth of B2394 (*ΔlivKHMGF ΔbrnQ*) and B2567 (*ΔlivKHMGF ΔbrnQ ΔyhjE*) strains on minimal medium supplemented with L-valine. Inactivation of *yhjE* increased the minimal inhibitory concentration of L-valine approximately fourfold, demonstrating that YhjE is directly involved in L-valine uptake (Figure 1C). Collectively, these phenotypic assays establish that YhjE significantly contributes to L-isoleucine and L-valine transport, but plays little, if any, role in L-leucine uptake.

**Figure 2:**
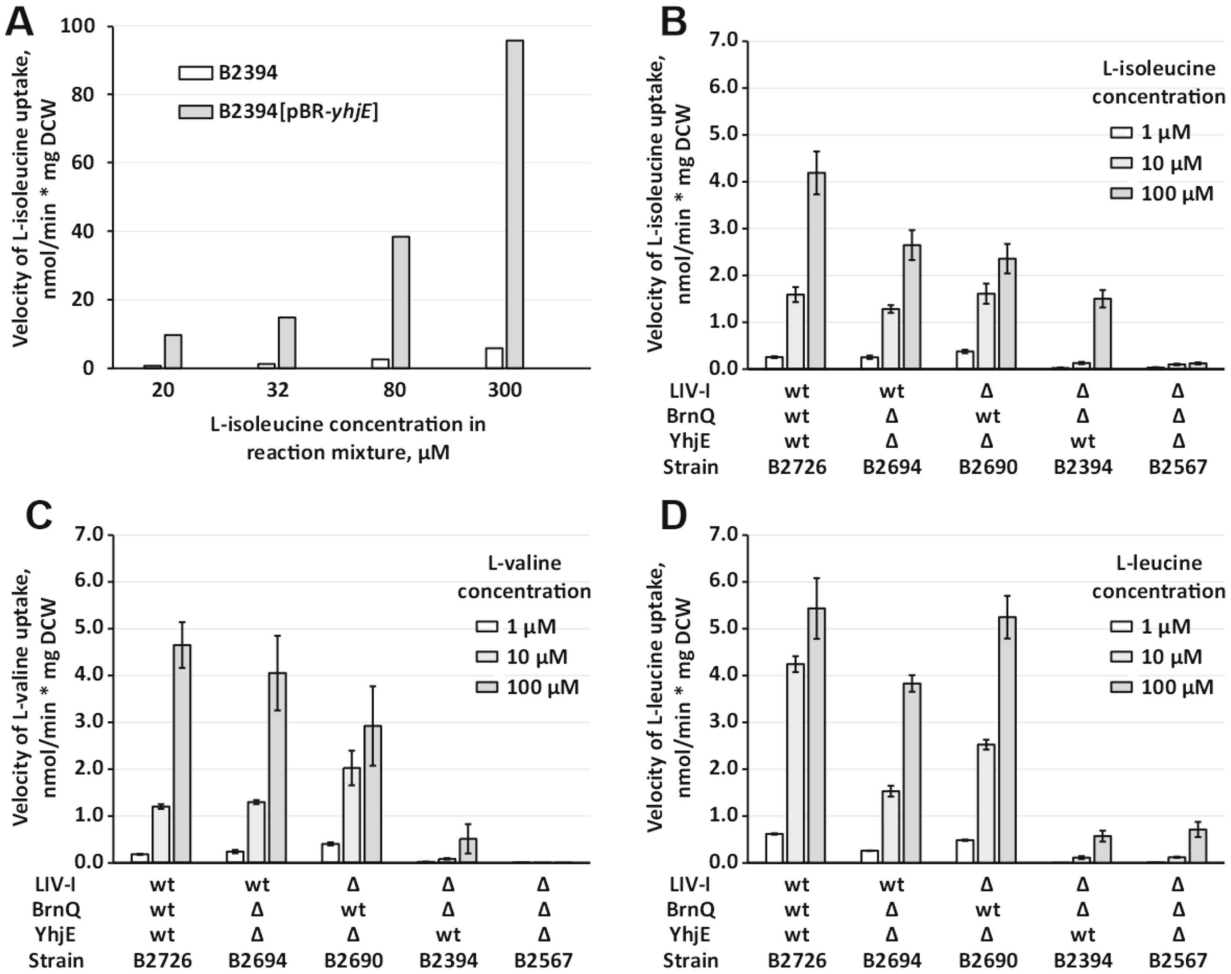
Analysis of YhjE activity toward L-isoleucine, L-valine, and L-leucine in comparison to the activities of LIV-I and BrnQ. The experiments were performed as described under “Amino acids uptake assay” in the Materials and Methods. The reaction time was 1 min. The concentrations of substrates are indicated in the figures. The values shown are the average of a single **(A)** or three **(B–D)** biological replicates; the error bars indicate SD. **(A)** Analysis of the effect of *yhjE* amplification due to the pBR-*yhjE* plasmid on the L-isoleucine-specific uptake activity of B2394 cells. **(B)** Comparison of L-isoleucine–specific activity of YhjE, LIV-I, and BrnQ. **(C)** Comparison of L-valine–specific activity of YhjE, LIV-I, and BrnQ. **(D)** Comparison of L-leucine–specific activity of YhjE, LIV-I, and BrnQ.

We next sought to directly assess the transport activity of YhjE toward individual branched-chain amino acids. To this end, we overexpressed *yhjE* from the pBR-*yhjE* plasmid in the *ΔlivKHMGF ΔbrnQ* background and measured L-isoleucine uptake. Amplification of *yhjE* led to a more than tenfold increase in uptake rate within the substrate concentration range of 20–300 μM, confirming that YhjE is indeed capable of mediating L-isoleucine import (Figure 2A).

To compare the relative contribution of YhjE to that of LIV-I and BrnQ, we measured uptake velocities of L-isoleucine, L-valine, and L-leucine in a strain that retained all the transport systems, and in double mutant strains carrying LIV-I, BrnQ, or YhjE as the sole carrier. These assays revealed three important features. First, the presence or absence of YhjE had no measurable effect on L-leucine transport activity, consistent with the phenotypic data indicating that YhjE is not a physiologically relevant L-leucine carrier (Figure 2D). Second, inactivation of *yhjE* essentially abolished L-isoleucine and L-valine uptake in the *ΔlivKHMGF ΔbrnQ* mutant background (Figures 2B,C). Third, although YhjE clearly contributed to L-isoleucine and L-valine uptake, its activity was lower than that of LIV-I or BrnQ and became prominent at higher substrate concentrations. Taken together, these results indicate that YhjE functions as a low-affinity permease specific for L-isoleucine and L-valine, while playing no significant role in L-leucine transport.

### 3.2 Kinetic parameters and substrate range of YhjE

To further characterize YhjE, we determined its kinetic parameters and substrate specificity. For this purpose, we used the B2933 strain carrying the native allele of *yhjE* on the pUC18 vector. Overexpression of YhjE in this background enabled reproducible transport measurements at elevated uptake rates. Kinetic analysis revealed that YhjE mediates import of both L-isoleucine and L-valine with comparable affinity: the apparent K_M_ values were 70.5 μM for L-isoleucine and 86.7 μM for L-valine. These affinities are markedly lower than those reported for the established BCAA permeases LIV-I and BrnQ, whose substrate K_M_ values fall in the submicromolar to low-micromolar range (Rahmanian et al., 1973). Thus, YhjE can be classified as a low-affinity transporter relative to the major BCAA carriers.

**Figure 3:**
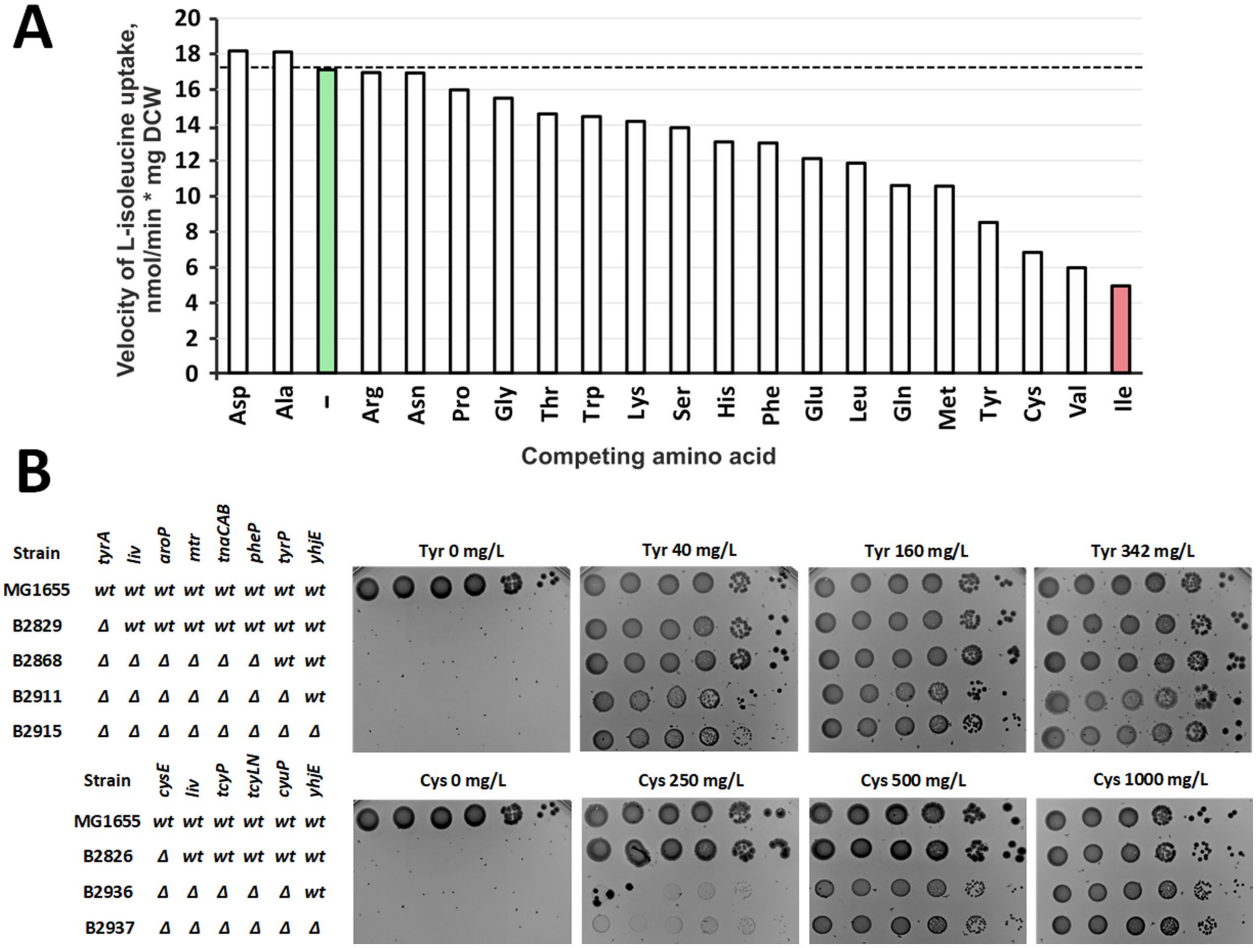
Analysis of the substrate range of YhjE. **(A)** The effect of a 30-fold molar excess of unlabeled amino acids on the L-isoleucine transport activity of YhjE. The experiments were performed using cells of the B2394[pBR-*yhjE*] strain. The reaction time was 1 min. The concentration of radiolabeled L-isoleucine was 50 μM. The results of a single biological replicate are shown. **(B)** Analysis of the effect of the *yhjE* mutation on the L-cysteine and L-tyrosine requirement. Phenotypic assays were performed on M9 minimal medium supplemented with 0.2% glucose and the indicated amino acid(s). “Cys” and “Tyr” denote L-cysteine and L-tyrosine, respectively. The genotype of each strain with respect to transport systems and auxotrophies is shown to the left of each panel; “*liv*” denotes *livKHMGF*. Upper and lower panels illustrate the effect of the *yhjE* mutation on the L-cysteine and L-tyrosine requirement, respectively.

We next examined the substrate selectivity of YhjE by testing the effect of 30-fold molar excess of unlabeled amino acids on uptake of radiolabeled L-isoleucine by the cells of the B2394[pBR-*yhjE*] strain. Among the 20 proteinogenic amino acids, only L-aspartate, L-asparagine, L-arginine, and L-alanine failed to inhibit YhjE activity (Figure 3A). All others reduced uptake to varying degrees, with the strongest effect observed for L-isoleucine itself, as expected. L-valine also markedly inhibited transport, in line with our previous demonstration that it serves as a YhjE substrate. Such inhibition of radiolabeled L-isoleucine uptake by unlabeled L-valine and L-isoleucine reflects direct competition for the same transport pathway, confirming that both amino acids are substrates of YhjE. Intriguingly, L-tyrosine and L-cysteine, which are structurally unrelated to branched-chain amino acids, produced pronounced inhibitory effects as well. To explore whether these amino acids might be additional physiological substrates of YhjE, we carried out phenotypic tests using auxotrophic strains defective in all known transporters for the corresponding amino acid (Figure 3B). The results indicated that YhjE inactivation does not alter the L-cysteine requirement and only slightly impairs the growth of the L-tyrosine-auxotrophic strain at L-tyrosine concentrations below 160 mg/L. Hence, YhjE is unlikely to be a dedicated permease for L-cysteine or L-tyrosine.

### 3.3 Structural basis of YhjE substrate specificity

To probe the molecular basis of the substrate specificity of YhjE—particularly its ability to transport L-isoleucine and L-valine but not L-leucine—we subjected the pBR-*yhjE* plasmid to *in vitro* hydroxylamine mutagenesis. The resulting library was introduced into the L-leucine-auxotrophic B2547 strain lacking LIV-I and BrnQ activities. Transformants were selected on minimal medium containing L-leucine at a concentration insufficient to support the growth of the isogenic B2547[pBR-*yhjE*] control, thereby enriching for variants in which *yhjE* mutations enhanced either overall activity or specificity toward L-leucine.

Sequencing of nine independently recovered mutant plasmids revealed six putative causative substitutions: P55S, S271F, T273I, T276I, V290M, and M359I. Specifically, the M359I and T276I substitutions were found in three independent clones each; the T273I and P55S were identified as the sole nonsynonymous mutations in corresponding clones. Finally, the S271F and V290M substitutions resided together in a single mutant allele. Nearly all recovered alleles carried multiple changes within the YhjE coding region (Supplementary Table S2). However, all the extra mutations were found to be synonymous. Finally, no mutations were found in the *yhjE* promoter region, excluding altered expression levels as an explanation. These observations supported the conclusion that the identified amino acid replacements underlie the gain of L-leucine transport activity.

**Figure 4:**
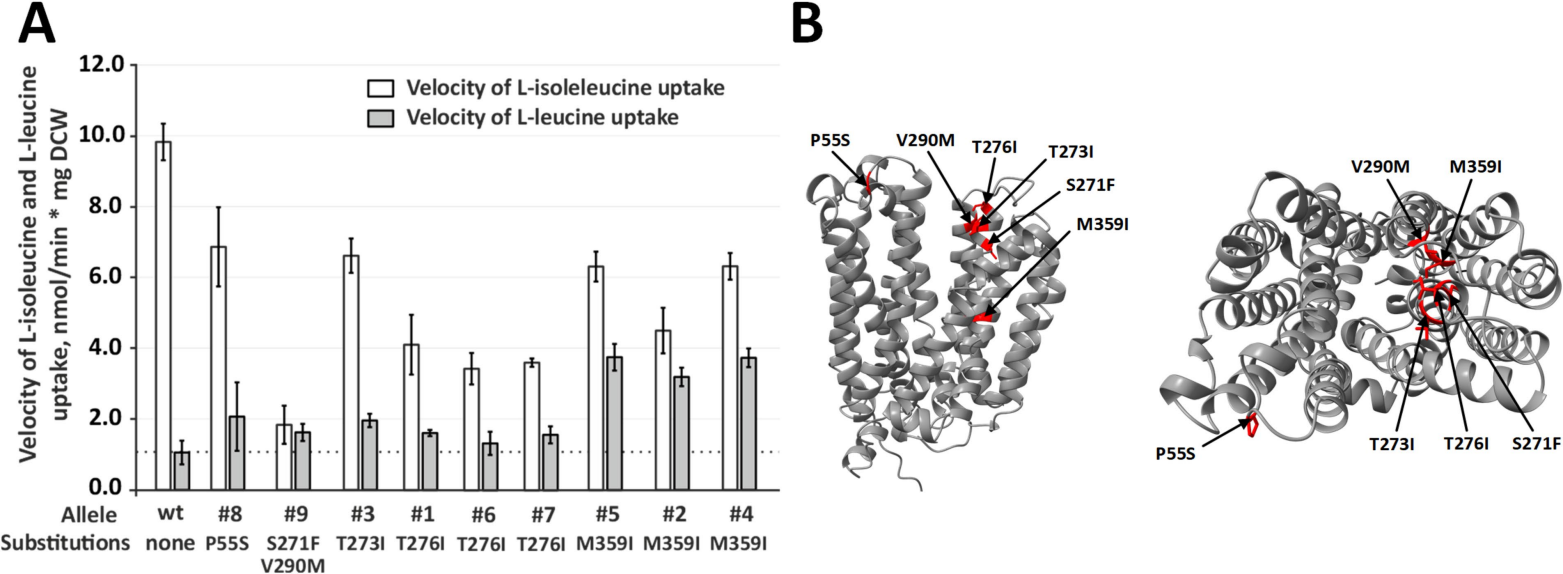
Analysis of the structural basis of L-isoleucine-over-L-leucine preference of YhjE. **(A)** Comparison of the activity of YhjE and its mutant derivatives toward L-isoleucine and L-leucine. The experiments were performed as described under “Amino acids uptake assay” in the Materials and Methods using the B2933 strain transformed with the pBR-*yhjE* plasmid and its mutant derivatives. The reaction time was 1 min. The concentrations of L-isoleucine and L-leucine were 50 μM. The values shown are the average of three biological replicates; the error bars indicate SD. A full set of substitutions in the indicated alleles can be found in Supplementary Table S2. **(B)** Tertiary structures of YhjE predicted by AlphaFold2 (Jumper et al., 2021). Red shading indicates mutated residues. The left and right panels represent the side and end views, respectively.

To determine whether the hydroxylamine-derived *yhjE* variants alter substrate preference rather than globally increasing transport capacity, we quantified uptake of L-isoleucine and L-leucine in the LIV-I/BrnQ-deficient strain B2933 carrying the mutant plasmids. In every case, L-leucine uptake was elevated relative to cells harboring the wild-type pBR-*yhjE*, whereas L-isoleucine uptake was concomitantly reduced (Figure 4A). The lowest effect was observed for the T276I substitution, while the YhjE M359I variant exhibited L-leucine transport activity more than twofold higher than the wild type YhjE permease. The coordinated increase in L-leucine transport accompanied by a decrease in L-isoleucine transport across all tested alleles demonstrates that the selected substitutions reprogram YhjE substrate specificity, rather than simply enhancing its overall activity.

Mapping the gain-of-function substitutions onto the topology of YhjE predicted by AlphaFold2 showed that, with the exception of P55S, all mutations (S271F, T273I, T276I, V290M, M359I) reside on the surface lining the putative translocation pathway. This spatial clustering provides a structural rationale for the observed specificity shift: local changes within the permeation corridor are expected to remodel the shape of the BCAA recognition pocket, thereby disfavoring L-isoleucine while accommodating L-leucine. By contrast, P55S maps to the external face of the protein and may influence an initial capture or gating step at the entry vestibule rather than the core binding pocket, offering a plausible route to increased L-leucine transport without globally enhancing activity (Figure 4B).

To rationalize these observations, we performed structure-guided docking followed by short molecular dynamics (MD) simulations on an AlphaFold-predicted model of YhjE (see Supplementary materials and Methods for protocols and Supplementary Results for detailed analyses). Docking converged on three recurrent ligand poses arranged along the putative permeation pathway: a surface Site 3 near the entry loop (around P55), a mid-channel Site 2 that acts as a selectivity checkpoint, and a deep Site 0 that forms a conserved “anchor” pocket. MD indicated that Sites 0 and 2 retain stable poses, whereas Site 3 behaves as a low-affinity capture site. In the wild-type protein, L-isoleucine is favored over L-leucine at Site 2, with selectivity supported by a high-occupancy H-bond to N178. By contrast, Site 0 exhibits a largely invariant H-bonding network (notably N77 and Y265) for both ligands, implying that discrimination occurs earlier along the pathway.

The gain-of-function substitutions map onto this framework via two distinct mechanisms. The S271F/V290M pair, located near Site 2, “softens” the selectivity filter: L-leucine adopts an alternative pose stabilized by a compensatory H-bond to T43, effectively erasing the wild-type energy gap between L-leucine and L-isoleucine at this checkpoint. In parallel, M359I enhances L-leucine capture at Site 3 by increasing entry-loop rigidity; notably, the deep M359I variant exerts this effect allosterically, reducing the mobility of the entry region. Together, these routes explain how discrete substitutions trade L-isoleucine preference for L-leucine transport without a global gain of function. For T273I and T276I, our docking and MD did not reveal consistent pose rearrangements or energetic signatures; accordingly, these variants were not pursued further in the computational analysis (see Supplementary Results).

### 3.4 Energetic coupling mechanism of YhjE-mediated transport

**Figure 5:**
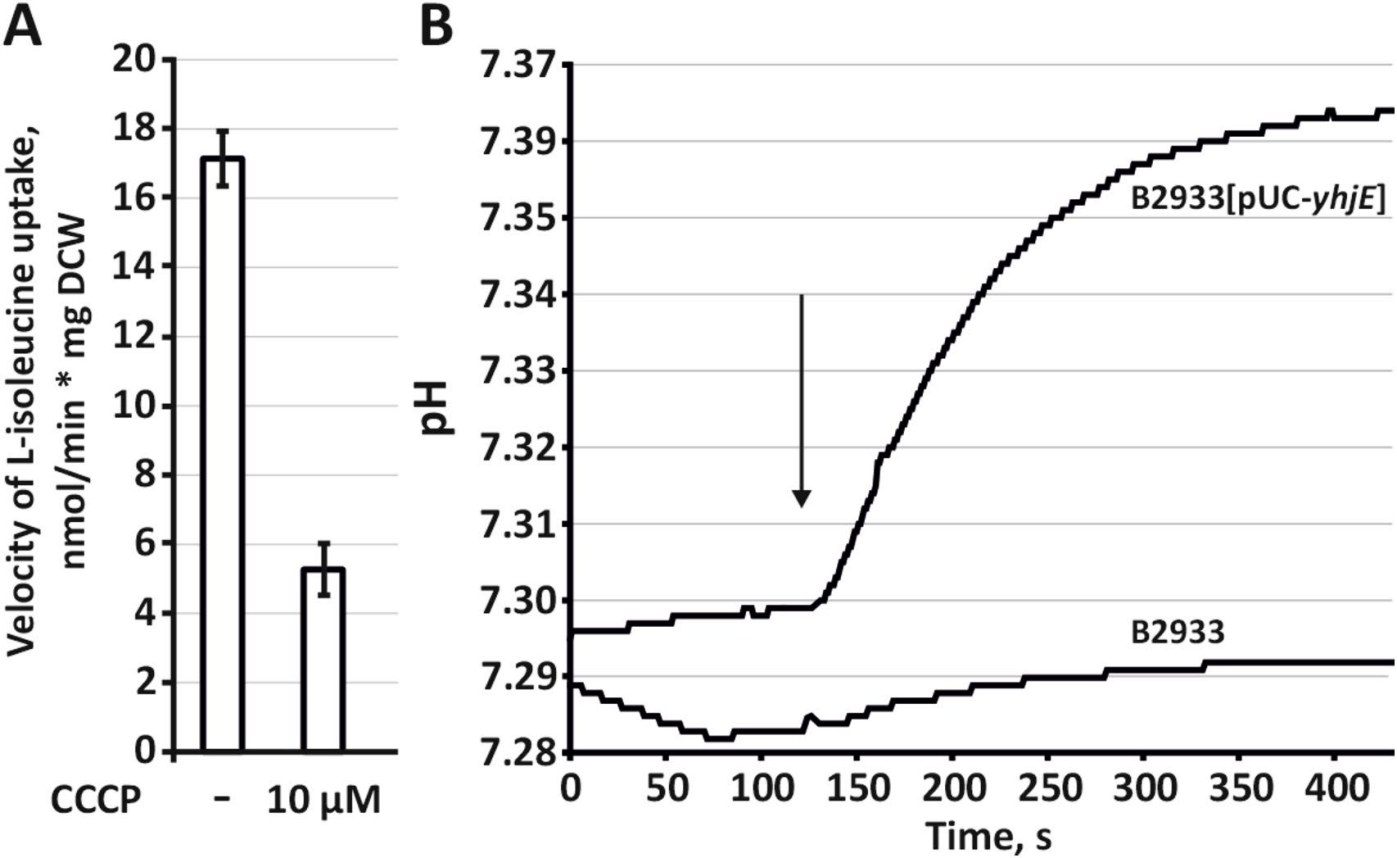
Analysis of the energetic coupling mechanism of L-isoleucine uptake via YhjE. **(A)** Effect of a 15-min preincubation of cells with the protonophore CCCP on the L-isoleucine uptake activity of YhjE. The experiment was performed as described under “Amino acids uptake assay” in the Materials and Methods using the B2567[pBR-*yhjE*] strain. The reaction time was 1 min. The concentration of L-isoleucine was 50 μM. The values shown are the average of three biological replicates; the error bars indicate SD. **(B)** pH change induced by L-isoleucine addition (indicated by a vertical arrow) in a suspension of the B2933[pUC-*yhjE*] strain overexpressing YhjE and the control B2933 strain. The experiments were performed as described under “Energetic coupling assay via pH measurement” in the Materials and Methods. The data shown are representative of an experiment performed with two biological replicates.

To elucidate the energetic coupling of BCAA transport via YhjE, we first assessed the effect of the protonophore CCCP on L-isoleucine uptake. Preincubation with CCCP markedly reduced the rate of L-[^3^H]isoleucine accumulation in the B2567[pBR-*yhjE*] strain, in which YhjE serves as the sole BCAA carrier (Figure 5A). Complementary pH-metry provided direct evidence for proton cotransport: addition of L-isoleucine triggered an immediate alkalinization of the external medium (consistent with proton influx into cells) in the YhjE-overexpressing strain, whereas no such response was detected in the isogenic control lacking YhjE activity (Figure 5B). Together, these observations demonstrate that YhjE mediates proton-motive-force–dependent symport.

### 3.5 Regulation of YhjE expression

**Figure 6:**
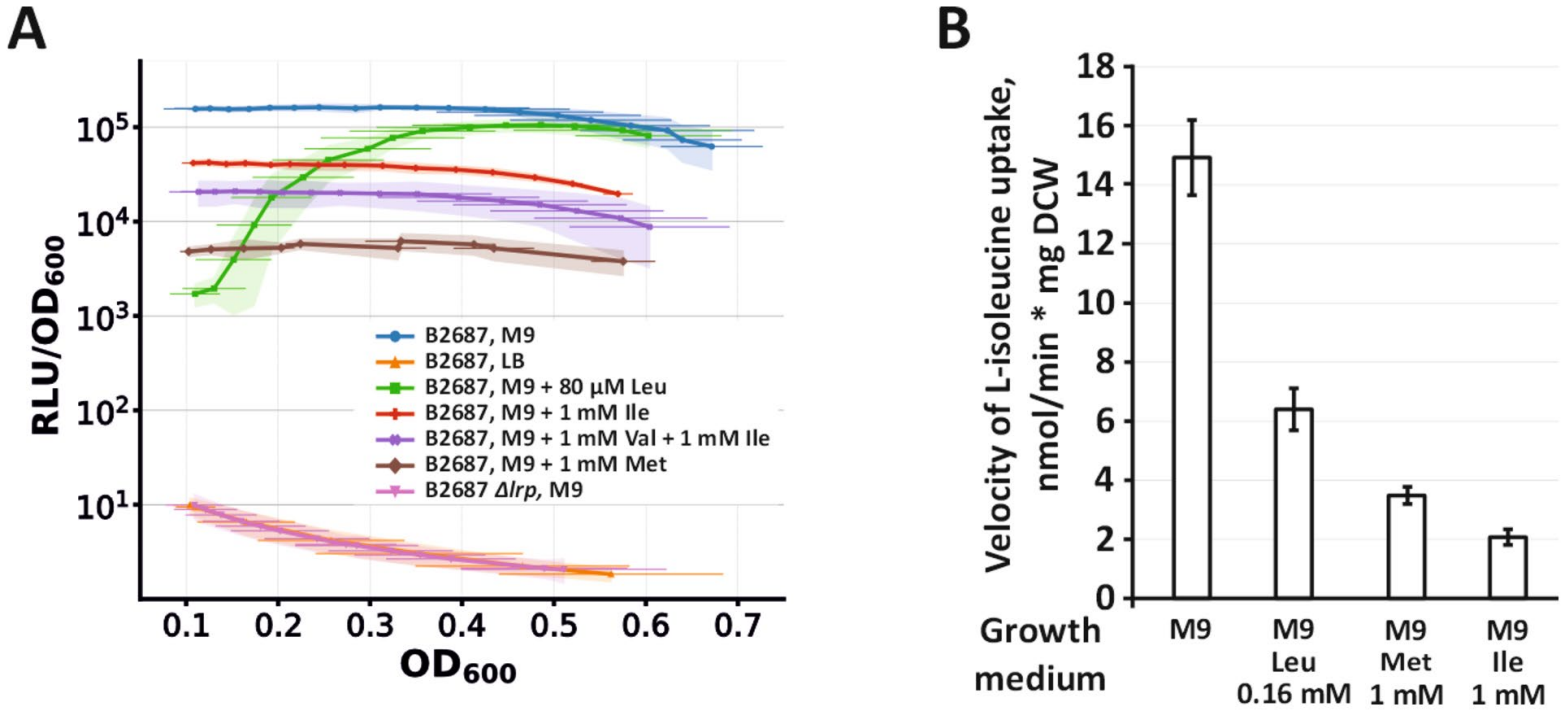
Analysis of the regulation of YhjE expression. **(A)** Quantification of *yhjE* promoter activity via bioluminescence measurement in strains carrying the P*_yhjE_*–*luxCDABE* transcriptional fusion and either the *lrp^wt^* or *Δlrp* allele. The values shown are the average of six independent biological replicates. Horizontal error bars and the filled area indicate the standard deviation for OD600 and RLU/OD600, respectively. The experiments were performed as described under “Measurement of *in vivo* luminescence and bacterial growth” in the Materials and Methods. The cells were cultured in either LB or M9 medium supplemented with 0.2% glucose and amino acids as indicated in the figure. The L-leucine concentration was reduced to 80 μM (compared to 1 mM for other amino acids) due to its toxicity. Thus, an apparent increase in bioluminescence under these conditions reflects L-leucine exhaustion. **(B)** Effect of L-leucine, L-isoleucine, and L-methionine addition to the growth medium on YhjE activity. The experiment was performed as described under “Amino acids uptake assay” in the Materials and Methods using the B2567[pBR-*yhjE*] strain. The reaction time was 1 min. The concentration of L-isoleucine in the reaction mixture was 50 μM. The values shown are the average of three biological replicates; the error bars indicate SD.

To delineate the regulatory logic of *yhjE* expression, we combined a chromosomal promoter–reporter assay with functional uptake measurements. First, we constructed a single-copy transcriptional fusion in which the native *yhjE* promoter drives expression of the *luxCDABE* operon of *Photorhabdus luminescens*. Reporter activity was strictly Lrp-dependent: inactivation of Lrp reduced luminescence by approximately three orders of magnitude, indicating that Lrp is essential for *yhjE* transcription. We then profiled the effect of all 20 amino acids on promoter output. Only three amino acids produced a significant response—L-leucine (as expected for an Lrp co-effector) and, unexpectedly, L-methionine and L-isoleucine—each causing marked repression of the *yhjE* promoter (Figure 6A). Consistent with promoter repression by these amino acids, growth in rich LB medium reduced reporter expression to a level comparable to that observed upon Lrp inactivation.

These transcriptional trends were mirrored at the level of transport activity. Cells of B2567[pBR-*yhjE*], in which YhjE serves as the sole BCAA carrier were cultivated in minimal M9 supplemented with either L-leucine, L-methionine, or L-isoleucine, then washed and assayed for L-isoleucine uptake in an amino acid–free reaction mixture. Pregrowth with any of the three amino acids substantially reduced YhjE-mediated uptake (Figure 6B). Together, these observations indicate that *yhjE* expression is downregulated by specific amino acids: L-leucine behaves consistently with its established role as an Lrp co-effector, while the inhibitory effects of L-methionine and L-isoleucine reveal additional, as yet unresolved layers of control (potentially Lrp-dependent or Lrp-independent).

## 4 Discussion

Transport of branched-chain amino acids (BCAAs) is at the crossroads of bacterial physiology, pathogenesis, and biotechnological performance. Uptake capacity and regulation determine growth in fluctuating environments, influence virulence traits in pathogens, and constrain flux in production strains. Despite decades of work and the identification of LIV-I/LS and LIV-II, the BCAA import repertoire of *Escherichia coli* has remained only partly resolved: reported activities have not been fully reconciled with genetic identities, and aspects of energetics and regulation have lacked definitive attribution. To address these gaps, we undertook an analysis of BCAA transport in *E. coli*. Within our study, independent observations made in parallel converged on the same locus, and through a set of orthogonal experiments we identified YhjE as a previously unassigned component of the import system. We then positioned YhjE within the existing network by combining genetics, phenotypic assays, direct transport measurements, kinetic analysis, energetic probing, promoter activity analysis, and targeted mutagenesis.

Previously, Khalfaoui-Hassani et al. reported that deletion of *yhjE* compromises growth when respiration is forced through the bo3 quinol oxidase and inferred a role for YhjE in metal homeostasis linked to bo_3_ biogenesis (Khalfaoui-Hassani et al., 2023). importantly, they did not demonstrate any metal-transport activity for YhjE. By contrast, our data evidently support the view that YhjE is a BCAA permease with a restricted substrate range, mediating uptake of L-isoleucine and L-valine but not L-leucine. Disruption of YhjE in a LIV-I/BrnQ-deficient background revealed a marked increase in the L-isoleucine requirement and heightened resistance to exogenous L-valine, whereas the L-leucine requirement was unchanged. Direct uptake assays demonstrated robust L-isoleucine and L-valine import attributable to YhjE, with no measurable contribution to L-leucine under the same conditions. Thus, bo_3_-linked phenotypes of *ΔyhjE* are more plausibly indirect metabolic consequences rather than evidence of direct metal transport, and the putative connection between YhjE and bo3 biogenesis/metal homeostasis will require further targeted investigation to resolve.

Kinetic characterization places YhjE in the low-affinity class relative to the canonical BCAA-specific systems: the apparent K_M_ values of 70.5 μM (L-isoleucine) and 86.7 μM (L-valine) are substantially higher than those reported or inferred for LIV-I and for BrnQ. Functionally, this suggests that YhjE contributes little at low substrate concentrations but becomes increasingly relevant as extracellular BCAA levels rise. This pattern is consistent with a division of labor in which high-affinity systems (e.g., LIV-I) dominate uptake under scarcity, whereas lower-affinity, higher-capacity transporters (e.g., YhjE) support flux at elevated substrate availability.

Mechanistically, YhjE operates as a proton-motive-force–dependent symporter: the protonophore CCCP sharply inhibited uptake, and real-time pH measurements reported proton influx upon L-isoleucine addition in a strain where YhjE is the sole BCAA carrier. This energetic profile aligns with its placement in the Metabolite: H + Symporter family.

At the regulatory level, *yhjE* transcription is strictly dependent on Lrp, and promoter activity is repressed by L-leucine; additional repression after pre-growth with L-methionine or L-isoleucine shows that the Lrp–leucine module alone does not account for promoter control. Together, these features indicate that YhjE expression is tuned to nutrient context and is downregulated when BCAAs are abundant—again consistent with a role as a conditional, low-affinity port of entry.

Targeted mutagenesis illuminates the narrow mutational target for altering YhjE specificity. Two gain-of-function substitutions, specifically, T276I and M359I, that confer activity toward L-leucine (each recovered three times in independent clones) underscore that only a limited set of sequence changes can achieve this outcome, and our screen likely captured most of them. Recurrent, nonrandom clustering of substitutions along the putative translocation pathway provides a structural rationale for specificity reprogramming. Docking and short MD simulations resolve two mechanistic routes consistent with these genetic observations: local “filter softening” at Site 2, exemplified by S271F/V290M, which allows L-leucine to adopt an alternative pose stabilized by a compensatory H-bond to T43 and thereby neutralizes the wild-type preference for L-isoleucine; and enhanced entry capture at Site 3, exemplified by the deep M359I substitution, which increases entry-loop rigidity and promotes initial L-leucine binding, with M359I acting allosterically from the protein core. Integrating mutagenesis, docking, and MD suggests the following specificity mechanism for YhjE: substrates are first caught at a low-affinity entry site (Site 3), then vetted at a mid-channel selectivity checkpoint (Site 2) that disfavors L-leucine, and finally anchored in a largely invariant deep pocket (Site 0) that does not itself provide discrimination. In this framework, specificity switching arises either by relaxing the Site 2 barrier (permitting L-leucine passage) or by strengthening initial capture at Site 3 so that L-leucine more readily engages the pathway.

Finally, the near-abolition of BCAA uptake in the *ΔlivKHMGF ΔbrnQ ΔyhjE* triple mutant at substrate concentrations up to 100 μM indicates that the combined activities of LIV-I, BrnQ, and YhjE account for physiologically relevant L-isoleucine and L-valine import under our conditions. Taken together, these findings argue against the existence of additional physiologically significant uptake systems for these amino acids in *E. coli* at typical extracellular levels. In light of its specificity and mechanism, we propose renaming *yhjE* to *ilvP* (L-isoleucine/L-valine permease).

## Data Availability

The raw data supporting the conclusions of this article will be made available by the authors, without undue reservation.

## References

[ref1] Adams M. D. Wagner L. M. Graddis T. J. Landick R. Antonucci T. K. Gibson A. L. . (1990). Nucleotide sequence and genetic characterization reveal six essential genes for the LIV-I and LS transport systems of *Escherichia coli*. J. Biol. Chem. 265, 11436–11443. doi: 10.1016/s0021-9258(19)38417-0, PMID: 2195019

[ref2] Antonucci T. K. Landick R. Oxender D. L. (1985). The leucine binding proteins of *Escherichia coli* as models for studying the relationships between protein structure and function. J. Cell. Biochem. 29, 209–216. doi: 10.1002/jcb.240290305, PMID: 4077929

[ref3] Badun G. A. Chernysheva M. G. (2023). Tritium thermal activation method. Features of application, modern achievements, and further development prospects. Radiochemistry 65, 185–197. doi: 10.1134/S1066362223020054

[ref4] Badun G. A. Chernysheva M. G. Ksenofontov A. L. (2012). Increase in the specific radioactivity of tritium-labeled compounds obtained by tritium thermal activation method. Radiochim. Acta 100, 401–408. doi: 10.1524/ract.2012.1926

[ref5] Bubnov D. M. Khozov A. A. Vybornaya T. V. Stepanova A. A. Molev S. V. Melkina O. E. . (2025). Multiple routes for non-physiological l-threonine uptake in *Escherichia coli* K-12. Front. Microbiol. 16:1579813. doi: 10.3389/fmicb.2025.1579813, PMID: 40248429 PMC12003319

[ref9001] Datsenko K. A. Wanner B. L. (2000). One-step inactivation of chromosomal genes in Escherichia coli K-12 using PCR products. Proceedings of the National Academy of Sciences of the United States of America 97, 6640–6645. doi: 10.1073/pnas.120163297 10829079 PMC18686

[ref9002] Dutta S. Corsi I. D. Bier N. Koehler T. M. (2022). BrnQ-Type Branched-Chain Amino Acid Transporters Influence Bacillus anthracis Growth and Virulence. mBio 13:e03640-21. doi: 10.1128/mbio.03640-21 35073743 PMC8787487

[ref6] Hoshino T. Kose K. Uratani Y. (1990). Cloning and nucleotide sequence of the gene braB coding for the sodium-coupled branched-chain amino acid carrier in *Pseudomonas aeruginosa* PAO. Mol. Gen. Genet. 220, 461–467. doi: 10.1007/BF00391754, PMID: 2111004

[ref7] Iaccarino M. Guardiola J. De Felice M. (1978). On the permeability of biological membranes. J. Membr. Sci. 3, 287–302. doi: 10.1016/S0376-7388(00)83028-8

[ref8] Jumper J. Evans R. Pritzel A. Green T. Figurnov M. Ronneberger O. . (2021). Highly accurate protein structure prediction with AlphaFold. Nature 596, 583–589. doi: 10.1038/s41586-021-03819-2, PMID: 34265844 PMC8371605

[ref9] Kaiser J. C. Heinrichs D. E. (2018). Branching out: alterations in bacterial physiology and virulence due to branched-chain amino acid deprivation. MBio 9:e01188–18. doi: 10.1128/mbio.01188-18 30181248 PMC6123439

[ref10] Kaiser J. C. Omer S. Sheldon J. R. Welch I. Heinrichs D. E. (2015). Role of BrnQ1 and BrnQ2 in branched-chain amino acid transport and virulence in *Staphylococcus aureus*. Infect. Immun. 83, 1019–1029. doi: 10.1128/IAI.02542-14, PMID: 25547798 PMC4333469

[ref11] Khalfaoui-Hassani B. Blaby-Haas C. E. Verissimo A. Daldal F. (2023). The *Escherichia coli* MFS-type transporter genes yhjE, ydiM, and yfcJ are required to produce an active bo3 quinol oxidase. PLoS One 18:e0293015. doi: 10.1371/journal.pone.0293015, PMID: 37862358 PMC10588857

[ref12] Khozov A. A. Bubnov D. M. Plisov E. D. Vybornaya T. V. Yuzbashev T. V. Agrimi G. . (2023). A study on L-threonine and L-serine uptake in *Escherichia coli* K-12. Front. Microbiol. 14:1151716. doi: 10.3389/fmicb.2023.1151716, PMID: 37025642 PMC10070963

[ref13] Koyanagi T. Katayama T. Suzuki H. Kumagai H. (2004). Identification of the LIV-I/LS system as the third phenylalanine transporter in *Escherichia coli* K-12. J. Bacteriol. 186, 343–350. doi: 10.1128/JB.186.2.343-350.2004, PMID: 14702302 PMC305776

[ref14] Ogawa-Miyata Y. Kojima H. Sano K. (2001). Mutation analysis of the feedback inhibition site of aspartokinase III of *Escherichia coli* K-12 and its use in L-threonine production. Biosci. Biotechnol. Biochem. 65, 1149–1154. doi: 10.1271/bbb.65.1149, PMID: 11440130

[ref15] Ohnishi K. Hasegawa A. Matsubara K. Date T. Okada T. Kiritani K. (1988). Cloning and nucleotide sequence of the brnQ gene, the structural gene for a membrane-associated component of the LIV-II transport system for branched-chain amino acids in *Salmonella typhimurium*. Jpn. J. Genet. 63, 343–357. doi: 10.1266/jjg.63.343, PMID: 3078876

[ref9003] Palace S. G. Proulx M. K. Lu S. Baker R. E. Goguen J. D. (2014). Genome-wide mutant fitness profiling identifies nutritional requirements for optimal growth of Yersinia pestis in deep tissue. mBio 5:e01385-14. doi: 10.1128/mBio.01385-14 25139902 PMC4147864

[ref9004] Park J. H. Lee K. H. Kim T. Y. Lee S. Y. (2007). Metabolic engineering of Escherichia coli for the production of l-valine based on transcriptome analysis and in silico gene knockout simulation. Proc Natl Acad Sci U S A 104, 7797–7802. doi: 10.1073/pnas.0702609104 17463081 PMC1857225

[ref9005] Pettersen E. F. Goddard T. D. Huang C. C. Meng E. C. Couch G. S. Croll T. I. et al. (2021). UCSF ChimeraX : Structure visualization for researchers, educators, and developers. Protein Science 30, 70–82. doi: 10.1002/pro.3943 32881101 PMC7737788

[ref16] Quay S. C. Oxender D. L. (1976). Regulation of branched-chain amino acid transport in *Escherichia coli*. J. Bacteriol. 127, 1225–1238. doi: 10.1128/jb.127.3.1225-1238.1976, PMID: 783137 PMC232915

[ref17] Rahmanian M. Claus D. R. Oxender D. L. (1973). Multiplicity of leucine transport systems in *Escherichia coli* K-12. J. Bacteriol. 116, 1258–1266. doi: 10.1128/jb.116.3.1258-1266.1973, PMID: 4584809 PMC246482

[ref18] Saier M. H. Reddy V. S. Tsu B. V. Ahmed M. S. Li C. Moreno-Hagelsieb G. (2016). The transporter classification database (TCDB): recent advances. Nucleic Acids Res. 44, D372–D379. doi: 10.1093/nar/gkv1103, PMID: 26546518 PMC4702804

[ref9006] Sambrook J. Fritsch E. F. Maniatis T. (1989). Molecular cloning: a laboratory manual. Cold spring harbor laboratory press.

[ref9007] Thomason L. C. Costantino N. Court D. L. (2007). E. coli genome manipulation by P1 transduction. Curr Protoc Mol Biol 79, 1.17.1–1.17.8. doi: 10.1002/0471142727.mb0117s79 18265391

[ref9008] Varadi M. Anyango S. Deshpande M. Nair S. Natassia C. Yordanova G. . (2022). AlphaFold Protein Structure Database: massively expanding the structural coverage of protein-sequence space with high-accuracy models. Nucleic Acids Research 50, D439–D444. doi: 10.1093/nar/gkab1061 34791371 PMC8728224

[ref19] Vyazmensky M. Zherdev Y. Slutzker A. Belenky I. Kryukov O. Barak Z. . (2009). Interactions between large and small subunits of different acetohydroxyacid synthase isozymes of *Escherichia coli*. Biochemistry 48, 8731–8737. doi: 10.1021/bi9009488, PMID: 19653643

[ref9009] Xie X. Xu L. Shi J. Xu Q. Chen N. (2012). Effect of transport proteins on L-isoleucine production with the L-isoleucine-producing strain Corynebacterium glutamicum YILW. J Ind Microbiol Biotechnol 39, 1549–1556. doi: 10.1007/s10295-012-1155-4 22733295

[ref20] Yamato I. Ohki M. Anraku Y. (1979). Genetic and biochemical studies of transport systems for branched-chain amino acids in *Escherichia coli*. J. Bacteriol. 138, 24–32. doi: 10.1128/jb.138.1.24-32.1979, PMID: 374366 PMC218233

