# Supplementary material for: Deciphering a missing piece of the branched-chain amino acids uptake puzzle: YhjE is an L-isoleucine and L-valine transporter in Escherichia coli K-12
Source: Front Microbiol. 2025 Dec 3;16:1727951. doi: 10.3389/fmicb.2025.1727951 (PMC12708883; doi:10.3389/fmicb.2025.1727951)
Supplement: Supplementary file 1 [file Table_1.DOCX]

Supplementary Table S1. Mutations identified in the yhjE alleles that confers L-valine resistance

| Amino acid substitution | G65L | F84L | P125L | G282E | V298G | G415R |
| --- | --- | --- | --- | --- | --- | --- |
| Codon substitution | CAG→CTG | TTT→TTG | CCG→CTG | GGG→GAG | GTT→GGT | GGG→AGG |

Supplementary Table S2. Mutations identified in the yhjE alleles that complement the L-leucine uptake defect

| Position of amino acid residue | 55 | 127 | 239 | 260 | 266 | 271 | 273 | 273 | 276 | 286 | 290 | 359 | 431 | 440 |
| --- | --- | --- | --- | --- | --- | --- | --- | --- | --- | --- | --- | --- | --- | --- |
| Amino acid residue in WT YhjE | Pro | Leu | Leu | Thr | Ile | Ser | Thr | Thr | Thr | Pro | Val | Met | Leu | Leu |
| Amino acid residue in mutant YhjE | Ser | Leu | Leu | Thr | Ile | Phe | Ile | Thr | Ile | Pro | Met | Ile | Leu | Leu |
| Mutant number |  |  |  |  |  |  |  |  |  |  |  |  |  |  |
| 1 |  |  |  |  |  |  |  |  |  |  |  |  |  |  |
| 2 |  |  |  |  |  |  |  |  |  |  |  |  |  |  |
| 3 |  |  |  |  |  |  |  |  |  |  |  |  |  |  |
| 4 |  |  |  |  |  |  |  |  |  |  |  |  |  |  |
| 5 |  |  |  |  |  |  |  |  |  |  |  |  |  |  |
| 6 |  |  |  |  |  |  |  |  |  |  |  |  |  |  |
| 7 |  |  |  |  |  |  |  |  |  |  |  |  |  |  |
| 8 |  |  |  |  |  |  |  |  |  |  |  |  |  |  |
| 9 |  |  |  |  |  |  |  |  |  |  |  |  |  |  |

*Red shading denotes putative causative mutations; green shading denotes synonymous mutations

# Supplementary Materials and Methods

## Preparation of molecular systems for simulations

Because no experimentally determined structure is available for the *E. coli* transporter YhjE, we used an AlphaFold3-predicted model of the wild-type protein as the starting template (Abramson et al., 2024). Point substitutions (P55S, T273I, T276I, S271F/V290M, and M359I) were introduced in PyMOL (Mutagenesis Wizard)(Schrödinger, LLC, 2015). L‑isoleucine and L‑leucine structures were obtained from PubChem (Kim et al., 2025). The protonation states of L-isoleucine and L-leucine were adjusted in PyMOL to create their zwitterionic forms. Ligand force-field parameters and topology files were generated with the CGenFF online service (Vanommeslaeghe et al., 2010, 2012; Vanommeslaeghe and MacKerell, 2012).

## Molecular docking

To identify prospective binding sites and generate starting poses for molecular dynamics (MD), we performed docking with AutoDock Vina (Trott and Olson, 2010; Eberhardt et al., 2021). The search space was defined as a cubic box (47 × 47 × 47 Å³) centred at the center of mass of the residues encompassing the experimentally selected substitutions so as to cover the entire putative translocation corridor. For each protein–ligand combination, 100 independent docking runs were executed. The resulting poses were clustered and the most consistent clusters—reproduced across all analyzed protein variants (WT and mutants)—were retained.

## Molecular dynamics simulations

All MD simulations were carried out with GROMACS 2025.3 (Van Der Spoel et al., 2005; Hess et al., 2008; Abraham et al., 2015). Protein, lipids and ions were described with CHARMM36m force-field (Huang et al., 2017); water was modelled with TIP3P (Jorgensen et al., 1983). Systems were assembled using CHARMM‑GUI (Jo et al., 2008): the protein was oriented according to OPM (Orientations of Proteins in Membranes (Lomize et al., 2012)), embedded into a 1-palmitoyl-2-oleoyl-sn-glycero-3-phosphoethanolamine (POPE) bilayer, solvated, and neutralized by adding Na⁺ and Cl⁻ ions to a physiological concentration of 150 mM.

Before running the simulations, each system underwent the multi-stage equilibration protocol provided by CHARMM-GUI. The protocol included energy minimization followed by a series of six short MD runs in the NVT and NPT ensembles, with progressively relaxed position restraints on the heavy atoms of the protein, lipids, and ligand. The temperature was maintained at 310 K using the V-rescale thermostat (Bussi et al., 2007), and the pressure at 1 bar using the C-rescale barostat (Bernetti and Bussi, 2020).

For each of the selected key systems, production simulations were performed in the NPT ensemble for 10 ns. The integration time step was 2 fs. Long-range electrostatics were treated using the particle-mesh Ewald (PME) method (Darden et al., 1993). Coordinates were saved every 10 ps for subsequent analysis.

## Trajectory analysis

Analyses were performed using built-in GROMACS tools and custom Python scripts based on MDAnalysis (Michaud‐Agrawal et al., 2011; Gowers et al., 2016) and MDTraj (McGibbon et al., 2014). Ligand–protein binding free‑energy (ΔG_bind) was estimated by MM/PBSA Molecular Mechanics/Poisson-Boltzmann Surface Area using the g_mmpbsa software (Kumari et al., 2014) on 901-frame sets extracted from the final, more stable segment of each trajectory.

The stability of the ligand pose within a binding site was assessed by computing the heavy-atom root-mean-square deviation (RMSD) of the ligand relative to its starting conformation, after aligning the trajectory to the protein Cα atoms. The conformational mobility of individual amino-acid residues was quantified by the root-mean-square fluctuations (RMSF) of their Cα atoms over the simulation.

Hydrogen bonds between the ligand and the protein were analyzed with the GROMACS hbond module. A hydrogen bond was considered present if the donor–acceptor distance was ≤ 3.5 Å and the acceptor–donor–hydrogen angle was ≤ 30°. For key interactions, we calculated their occupancy, defined as the percentage of simulation time (frames) during which the bond persisted.

# Supplementary Results

**3.1. Molecular docking reveals a hierarchical series of binding sites along the translocation pathway**

To identify potential ligand–transporter interaction regions in YhjE, we performed large-scale molecular docking. Clustering of docking poses yielded four recurrent clusters; three of these—hereafter designated Site 0, Site 2, and Site 3—were consistently recovered across the majority of analyzed systems (WT and mutants with both ligands) and were retained for further analysis (Supplementary Figure S1). Site 1 showed markedly lower reproducibility and was therefore excluded from subsequent analyses. Site 2 also lacked consistent reproducibility across all systems; however, it was retained for analysis because it was predominantly observed in the WT and S271F/V290M variants and is located near the corresponding substitutions.


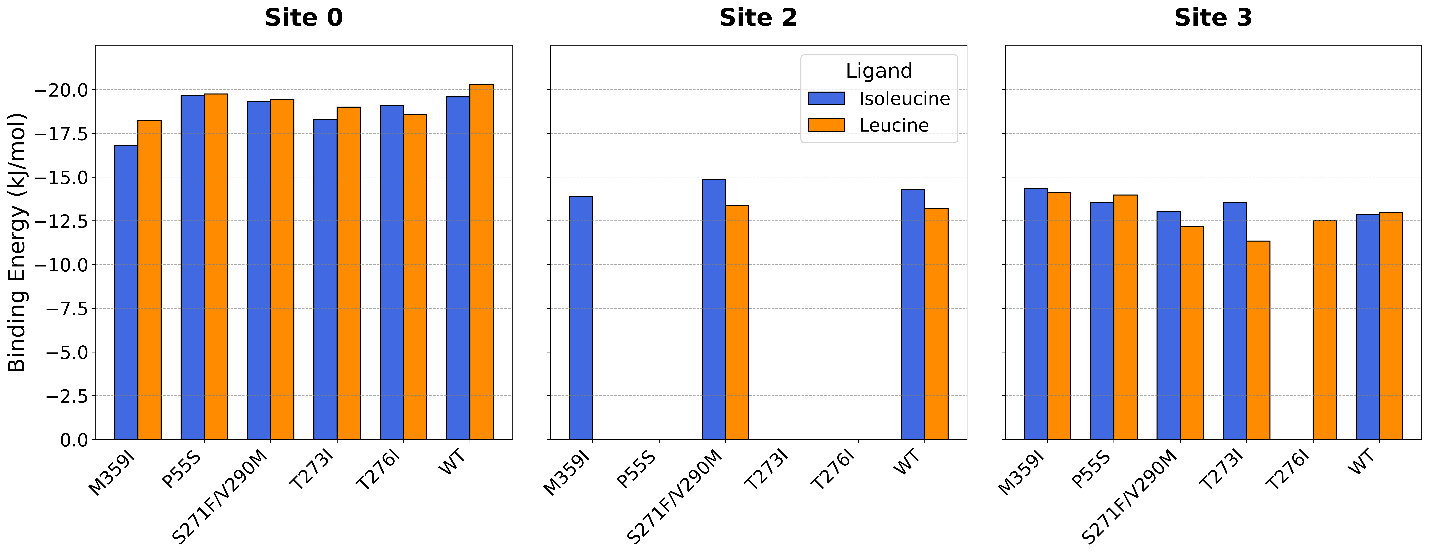


Supplementary Figure S1. Binding energies of L-leucine and L-isoleucine in predicted binding sites. Binding energies (kJ/mol) were calculated from molecular docking for L-isoleucine (blue) and L-leucine (orange) in the wild-type (WT) YhjE and five different mutants. The comparison is shown across three putative binding sites identified.

The identified sites delineate a clear path leading from the extracellular milieu to the protein core (Supplementary Figure S2). Site 3 is located on the protein surface, in proximity to a flexible loop around residue P55, and presumably functions as the primary recognition site. Site 2 is situated in the middle of the translocation pore, while Site 0 resides in the deepest, most hydrophobic pocket at the center of the protein globule, forming a cavity.


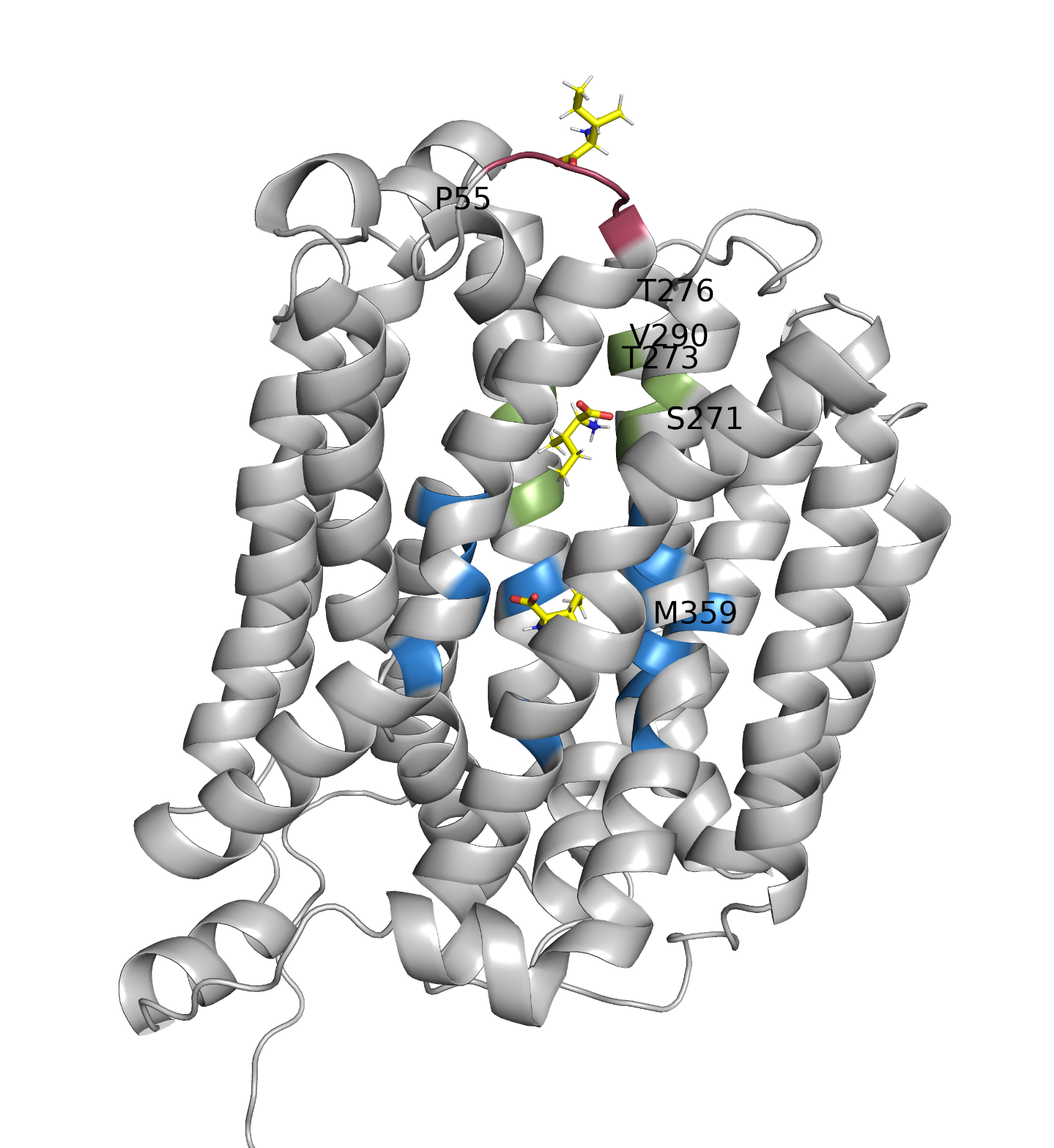


Supplementary Figure S2. Location of key mutations and predicted binding sites in the YhjE structure. Protein residues are color-coded based on their proximity to the binding sites: red indicates the surface entry site (Site 3, P55), green marks the central pore (Site 2, S271, T273, T276, and V290), and blue corresponds to the deep inner pocket (Site 0, M359). The docked ligand (L-isoleucine) is shown as yellow sticks.

To test the hypothesis of an energetic gradient along this path, we carried out targeted docking in which the initial ligand position was restricted to pose regions previously found in Sites 3 or 2. In these simulations, regardless of the starting position, the ligand invariably relaxed to the most energetically favourable state in the central Site 0, which is consistent with small differences among starting poses located near an energetically favourable route into the transporter. These data suggest that the identified sites represent local free-energy minima on an energy landscape, forming an “energy funnel” that guides substrate movement toward the protein center.

To further verify the docking results and to investigate in detail the atomic mechanisms underlying changes in substrate specificity, we performed molecular-dynamics simulations, primarily to determine ligand stability in each site. Owing to the substantial computational cost, a representative subset of systems was selected for this stage of the analysis, including the wild-type protein (WT) and mutants whose substitutions were localized in the immediate vicinity of the most consistently detected binding sites (P55S, M359I, S271F/V290M). This approach allowed us to focus on testing the most plausible hypotheses regarding the mechanisms of action of these mutations in Sites 0, 2, and 3. Mutants T273I and T276I, for which the preliminary docking analysis did not allow us to formulate unambiguous hypotheses about their mode of action, were not considered in subsequent stages of this part of the study.

## Quantitative assessment of mutational effects on ligand affinity in key sites

To quantify binding thermodynamics, the binding free energy was estimated by the MM/PBSA method. It should be noted that a 10-ns simulation does not allow reliable inference of exact numerical values; however, the obtained data can be used to evaluate overall trends. Comparing energies for the wild-type protein showed that it exhibits somewhat higher affinity for its native substrate, L-isoleucine, relative to L-leucine (Supplementary Figure S3). The most pronounced difference was observed in Site 2, indicating that this region may serve as the principal “selectivity filter,” imposing a high energetic barrier to the passage of the noncognate ligand.


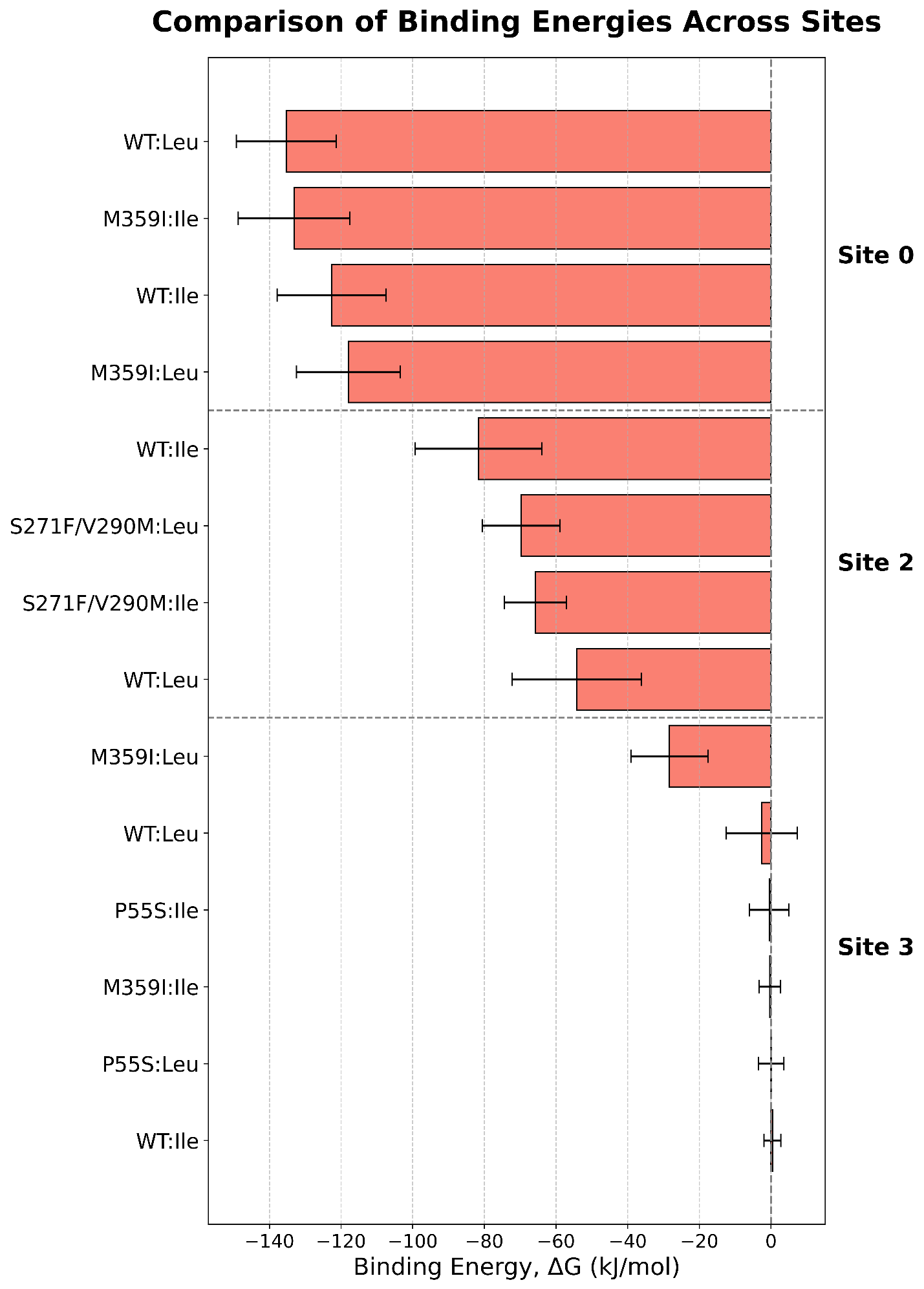


Supplementary Figure S3. Comparison of binding free energies for selected YhjE systems. The binding free energy (ΔG, in kJ/mol) of L-isoleucine (Ile) and L-leucine (Leu) to wild-type (WT) YhjE and selected mutants (M359I, S271F/V290M, P55S) in the three binding sites. Energies were estimated using the MM/PBSA method on trajectories from 10 ns molecular dynamics simulations. Error bars represent the standard deviation of the energy values over trajectory frames.

Analysis of mutant systems revealed two distinct adaptive mechanisms. The double mutation S271F/V290M, located in proximity to Site 2, led to an almost complete elimination of the difference in binding energies between L-leucine and L-isoleucine in this site. This indicates that the mutant acquires a new function by effectively “switching off” the native selectivity filter. Notably, the strongest stabilizing effect on binding in Site 3 was produced by the deep M359I mutation, which points to the presence of long-range allosteric communication between the protein core and its extracellular entry.

## Dynamic basis of the change in substrate specificity

Analysis of MD trajectories revealed dynamic processes underlying the observed energetic changes.

## 3.3.1. Binding stability

RMSD calculations confirmed that Sites 0 and 2 are stable binding sites in which ligands maintain a persistent pose throughout the simulations (RMSD < 0.1 nm). By contrast, Site 3 is characterized as a low-affinity region: in all simulations, the ligands exhibited high mobility and dissociated from this site (RMSD > 1), which was also confirmed by visual inspection of the trajectories.

## 3.3.2. Reorganization of the hydrogen-bond network

Detailed hydrogen bond analysis across trajectories (Supplementary Figure S4) showed that wild-type selectivity in Site 2 is determined by the formation of a highly occupied (91.7%) hydrogen bond between residue N178 and the amino group of L-isoleucine (Supplementary Figure S5B), which occurs much less frequently upon binding L-leucine (35.0%). In the S271F/V290M double-mutant system with L-leucine, we observed the formation of a new, compensatory, and very stable hydrogen bond with residue T43 (93.7% occupancy), as well as an increased frequency of bonding with N178 (Supplementary Figure S5B). This likely indicates that the reduction of the energetic barrier in the putative selectivity filter in this region is achieved by providing the ligand with an alternative stabilization point within the pocket.


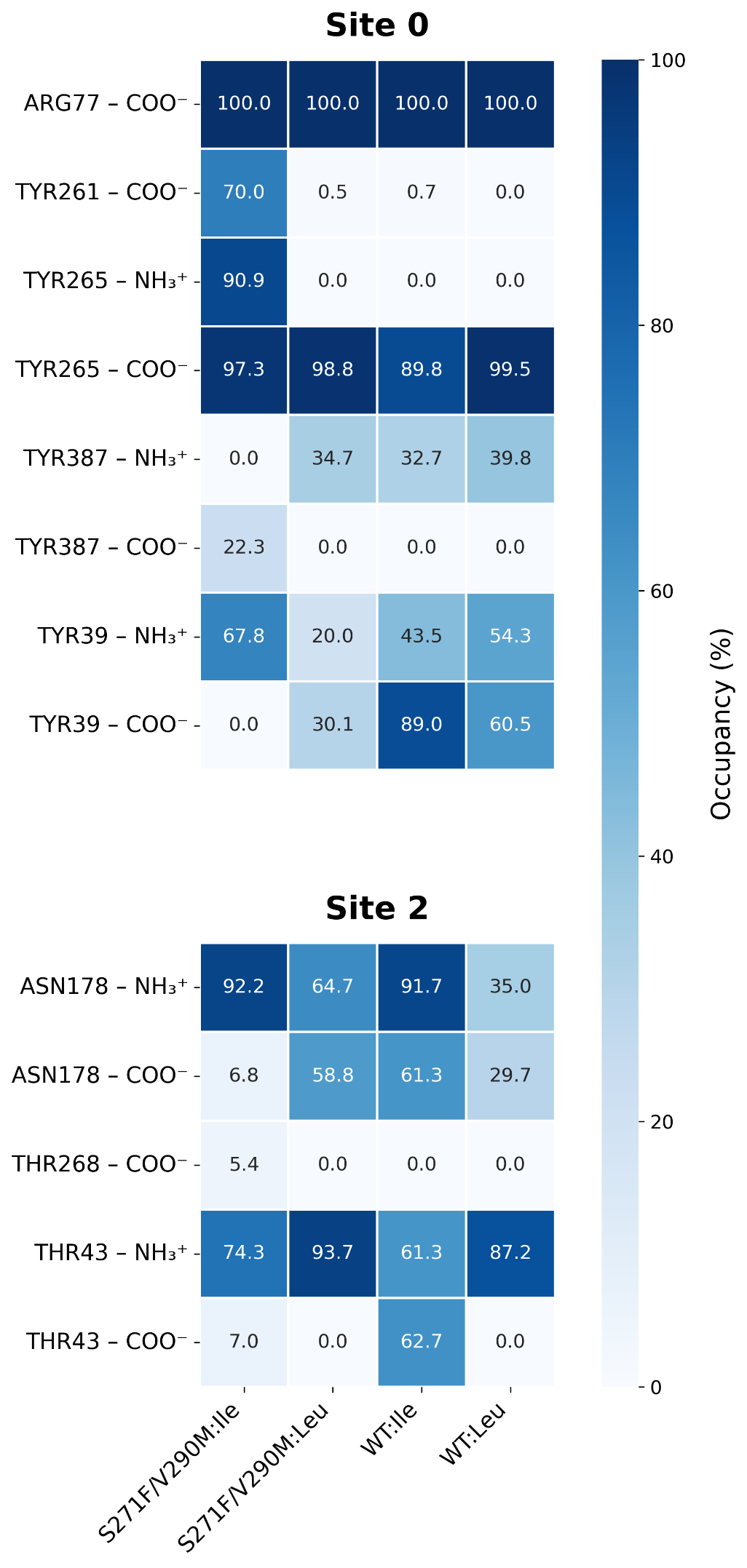


Supplementary Figure S4. Hydrogen bond occupancy in binding Sites 0 and 2. A heatmap showing the occupancy (%) of key hydrogen bonds between the ligand's carboxylate (COO^-^) or amino (NH3^+^) groups and protein residues. Occupancy, defined as the percentage of simulation time the bond was present, was calculated from molecular dynamics trajectories. The analysis compares the wild-type (WT) protein and the S271F/V290M double mutant when bound to either L-isoleucine (Ile) or L-leucine (Leu).

In the central Site 0, we identified a conserved and highly stable hydrogen-bond network dominated by interactions between the ligand carboxylate and the side chains of residues R77 (occupancy > 99%) and Y265 (occupancy > 90%) (Supplementary Figure S5A). This interaction network was observed in all analyzed systems (WT and mutants, with both ligands), supporting the role of Site 0 as a universal, high-affinity “anchor” for the substrate at the final stage before translocation. Interestingly, in the wild type we observed a higher occupancy of the bond with Y265 for L-leucine compared with L-isoleucine, which correlates with a more favourable MM/PBSA binding energy for L-leucine in this site, despite the lack of effective transport of this amino acid. This observation supports the hypothesis that the key selective barriers are located at earlier stages of ligand progression along the channel.


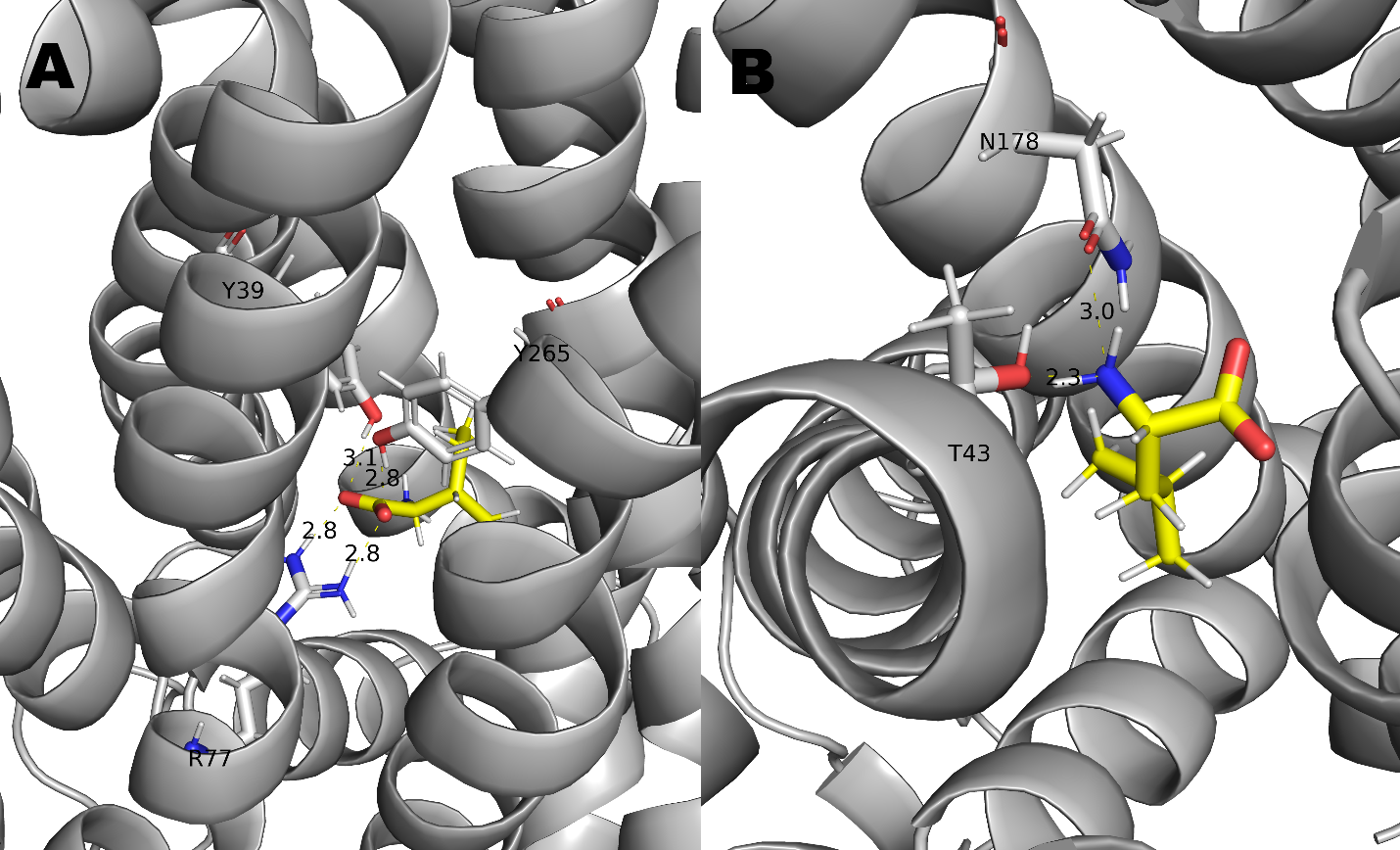


Supplementary Figure S5. Detailed view of the hydrogen-bond network in YhjE binding sites. Structural representation of key ligand-protein interactions. (A) The stable hydrogen bond network in the central binding site (Site 0), showing interactions of the ligand (yellow sticks) with residues R77, Y265, and Y39. (B) Key interactions within the selectivity filter (Site 2), highlighting hydrogen bonds with N178 and T43. Hydrogen bonds are shown as dashed lines, with distances in angstroms (Å).

## Modulation of conformational mobility

Analysis of root-mean-square fluctuations (RMSF) revealed distinct strategies by which mutations influence protein dynamics (Supplementary Figure S6). The P55S mutation, which enhances binding efficiency in the surface Site 3, caused a marked increase in rigidity (decrease in RMSF) in the entry-loop region (residues 55–57) and adjacent α-helical segments. Notably, the M359I mutation, located deep within the protein, induced a similar effect. This fact provides direct evidence for an allosteric communication pathway through which conformational changes in the protein core are transmitted to the periphery, modulating the dynamics of the entry site. These data suggest a hypothesis of “entry-site strengthening” and attest to the existence of an allosteric route by which conformational changes in the protein’s central region are conveyed to its periphery, modulating the dynamics of the region critical for ligand capture.

Interestingly, in both M359I and P55S mutants we observed not only a decrease in the conformational mobility of the loop in Site 3 (residue Q56), but also a substantial decrease in the mobility of residue R132. This region likely underpins conformational coupling between the local geometry of Site 3 and the structure of the protein deeper within its hydrophobic core.

By contrast, the S271F/V290M double mutation employs a second, alternative strategy based on increasing local plasticity. The double substitution S271F/V290M led to loosening of the structure in the Site 2 region. This change apparently constitutes a prerequisite for L-leucine to adopt a new conformation and form a compensatory hydrogen bond with residue T43, which is inaccessible in the more rigid wild-type structure. Importantly, analysis of clustered ΔRMSF profiles showed that mobility changes are not localized exclusively at the sites of substitution but are distributed over functionally connected yet spatially distant parts of the structure. This indicates that the observed adaptation is achieved not by directly introducing new interactions but through fine-tuning of local conformational ensembles. These rearrangements alter the orientation and availability of key residues along the entire channel, ultimately facilitating the passage of the new substrate.


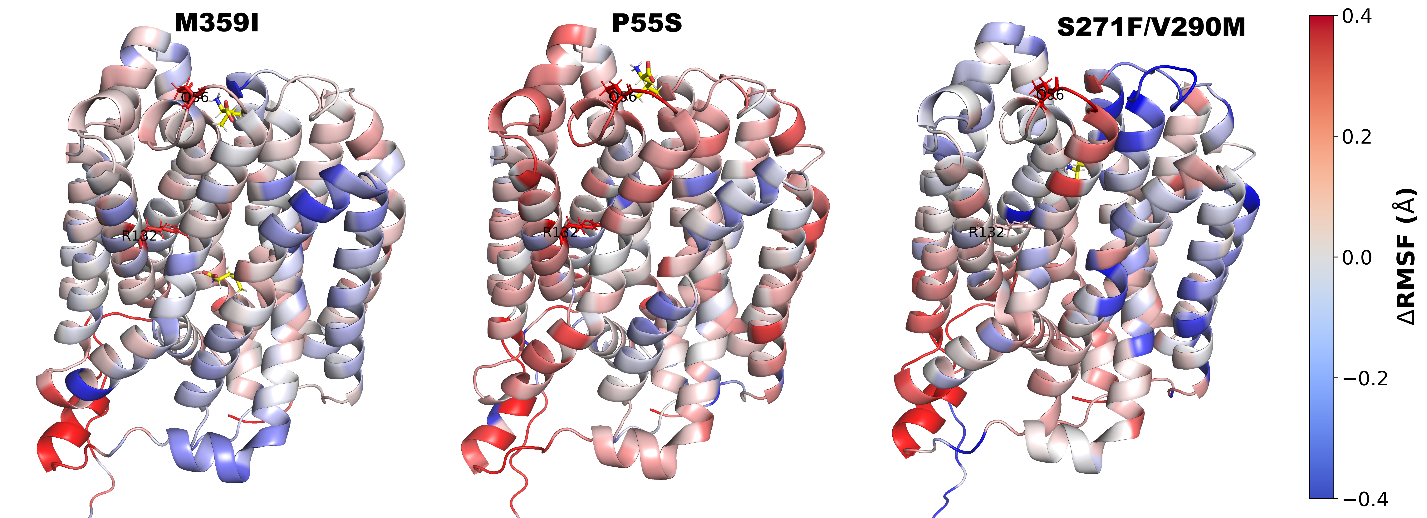


Supplementary Figure S6. Changes in protein conformational mobility induced by mutations. The YhjE protein structure is colored according to the difference in root-mean-square fluctuation (ΔRMSF) between the mutant and the wild-type protein. Red regions indicate increased mobility (flexibility) in the mutant, while blue regions indicate decreased mobility (rigidification). The distinct dynamic effects of the M359I, P55S, and S271F/V290M mutations are compared.

As a conclusion, it was established that the native protein likely possesses a multistage selectivity system in which primary ligand binding at Site 3 and an energetic barrier in the central portion of the translocation pore (Site 2), defined by interaction with residue N178, play important roles.

Analysis of the investigated mutations showed that adaptation to a new substrate, L-leucine, is achieved not via a single unified mechanism but through several distinct yet functionally convergent strategies aimed at modulating the protein’s energetic and dynamic landscape. The mechanisms identified include:

1. Reduction of the energetic barrier of the selectivity filter. The S271F/V290M double mutation induces a local increase in conformational plasticity in Site 2, allowing L-leucine to form an alternative, compensatory hydrogen bond with residue T43, effectively neutralizing the native energetic barrier;
2. Decrease in conformational mobility of the entry site. The P55S and M359I mutations enhance the efficiency of initial L-leucine capture in the surface Site 3. P55S acts locally by increasing the rigidity of the entry loop, whereas the deep M359I mutation achieves the same effect via long-range allosteric regulation, transmitting a conformational signal from the protein core to its periphery.

# Supplementary References

Abraham, M. J., Murtola, T., Schulz, R., Páll, S., Smith, J. C., Hess, B., et al. (2015). GROMACS: High performance molecular simulations through multi-level parallelism from laptops to supercomputers. *SoftwareX* 1–2, 19–25. doi: 10.1016/j.softx.2015.06.001

Abramson, J., Adler, J., Dunger, J., Evans, R., Green, T., Pritzel, A., et al. (2024). Accurate structure prediction of biomolecular interactions with AlphaFold 3. *Nature* 630, 493–500. doi: 10.1038/s41586-024-07487-w

Bernetti, M., and Bussi, G. (2020). Pressure control using stochastic cell rescaling. *The Journal of Chemical Physics* 153, 114107. doi: 10.1063/5.0020514

Bussi, G., Donadio, D., and Parrinello, M. (2007). Canonical sampling through velocity rescaling. *The Journal of Chemical Physics* 126, 014101. doi: 10.1063/1.2408420

Darden, T., York, D., and Pedersen, L. (1993). Particle mesh Ewald: An *N* ⋅log( *N* ) method for Ewald sums in large systems. *The Journal of Chemical Physics* 98, 10089–10092. doi: 10.1063/1.464397

Eberhardt, J., Santos-Martins, D., Tillack, A. F., and Forli, S. (2021). AutoDock Vina 1.2.0: New Docking Methods, Expanded Force Field, and Python Bindings. *J. Chem. Inf. Model.* 61, 3891–3898. doi: 10.1021/acs.jcim.1c00203

Gowers, R., Linke, M., Barnoud, J., Reddy, T., Melo, M., Seyler, S., et al. (2016). MDAnalysis: A Python Package for the Rapid Analysis of Molecular Dynamics Simulations., (Austin, Texas), 98–105. doi: 10.25080/Majora-629e541a-00e

Hess, B., Kutzner, C., Van Der Spoel, D., and Lindahl, E. (2008). GROMACS 4: Algorithms for Highly Efficient, Load-Balanced, and Scalable Molecular Simulation. *J. Chem. Theory Comput.* 4, 435–447. doi: 10.1021/ct700301q

Huang, J., Rauscher, S., Nawrocki, G., Ran, T., Feig, M., De Groot, B. L., et al. (2017). CHARMM36m: an improved force field for folded and intrinsically disordered proteins. *Nat Methods* 14, 71–73. doi: 10.1038/nmeth.4067

Jo, S., Kim, T., Iyer, V. G., and Im, W. (2008). CHARMM‐GUI: A web‐based graphical user interface for CHARMM. *J Comput Chem* 29, 1859–1865. doi: 10.1002/jcc.20945

Jorgensen, W. L., Chandrasekhar, J., Madura, J. D., Impey, R. W., and Klein, M. L. (1983). Comparison of simple potential functions for simulating liquid water. *The Journal of Chemical Physics* 79, 926–935. doi: 10.1063/1.445869

Kim, S., Chen, J., Cheng, T., Gindulyte, A., He, J., He, S., et al. (2025). PubChem 2025 update. *Nucleic Acids Research* 53, D1516–D1525. doi: 10.1093/nar/gkae1059

Kumari, R., Kumar, R., Open Source Drug Discovery Consortium, and Lynn, A. (2014). *g_mmpbsa* —A GROMACS Tool for High-Throughput MM-PBSA Calculations. *J. Chem. Inf. Model.* 54, 1951–1962. doi: 10.1021/ci500020m

Lomize, M. A., Pogozheva, I. D., Joo, H., Mosberg, H. I., and Lomize, A. L. (2012). OPM database and PPM web server: resources for positioning of proteins in membranes. *Nucleic Acids Research* 40, D370–D376. doi: 10.1093/nar/gkr703

McGibbon, R. T., Beauchamp, K. A., Schwantes, C. R., Wang, L.-P., Hernández, C. X., Herrigan, M. P., et al. (2014). MDTraj: a modern, open library for the analysis of molecular dynamics trajectories. doi: 10.1101/008896

Michaud‐Agrawal, N., Denning, E. J., Woolf, T. B., and Beckstein, O. (2011). MDAnalysis: A toolkit for the analysis of molecular dynamics simulations. *J Comput Chem* 32, 2319–2327. doi: 10.1002/jcc.21787

Schrödinger, LLC (2015). The PyMOL Molecular Graphics System, Version 3.0.

Trott, O., and Olson, A. J. (2010). AutoDock Vina: Improving the speed and accuracy of docking with a new scoring function, efficient optimization, and multithreading. *J Comput Chem* 31, 455–461. doi: 10.1002/jcc.21334

Van Der Spoel, D., Lindahl, E., Hess, B., Groenhof, G., Mark, A. E., and Berendsen, H. J. C. (2005). GROMACS: Fast, flexible, and free. *J Comput Chem* 26, 1701–1718. doi: 10.1002/jcc.20291

Vanommeslaeghe, K., Hatcher, E., Acharya, C., Kundu, S., Zhong, S., Shim, J., et al. (2010). CHARMM general force field: A force field for drug‐like molecules compatible with the CHARMM all‐atom additive biological force fields. *J Comput Chem* 31, 671–690. doi: 10.1002/jcc.21367

Vanommeslaeghe, K., and MacKerell, A. D. (2012). Automation of the CHARMM General Force Field (CGenFF) I: Bond Perception and Atom Typing. *J. Chem. Inf. Model.* 52, 3144–3154. doi: 10.1021/ci300363c

Vanommeslaeghe, K., Raman, E. P., and MacKerell, A. D. (2012). Automation of the CHARMM General Force Field (CGenFF) II: Assignment of Bonded Parameters and Partial Atomic Charges. *J. Chem. Inf. Model.* 52, 3155–3168. doi: 10.1021/ci3003649
